# Divergent and Compensatory Effects of BMP2 and BMP4 on the VSMC Phenotype and BMP4’s Role in Thoracic Aortic Aneurysm Development

**DOI:** 10.3390/cells13090735

**Published:** 2024-04-24

**Authors:** Daniel Klessinger, Argen Mamazhakypov, Sophie Glaeser, Ramona Emig, Remi Peyronnet, Lena Meier, Kora Proelss, Katia Marenne, Christian Smolka, Sebastian Grundmann, Franziska Pankratz, Philipp R. Esser, Martin Moser, Qian Zhou, Jennifer S. Esser

**Affiliations:** 1 Department of Cardiology and Angiology, University Heart Center Freiburg-Bad Krozingen, Medical Center-University of Freiburg, Faculty of Medicine, University of Freiburg, 79106 Freiburg im Breisgau, Germanychristian.smolka@uniklinik-freiburg.de (C.S.); sebastian.grundmann@uniklinik-freiburg.de (S.G.); franziska.pankratz@uniklinik-freiburg.de (F.P.); martin.moser@uniklinik-freiburg.de (M.M.); qian.zhou@usb.ch (Q.Z.); 2 Institute of Experimental and Clinical Pharmacology and Toxicology, Faculty of Medicine, University of Freiburg, 79104 Freiburg im Breisgau, Germany; argen.mamazhakypov@pharmakol.uni-freiburg.de; 3 Institute for Experimental Cardiovascular Medicine, University Heart Center Freiburg-Bad Krozingen, Medical Center-University of Freiburg, Faculty of Medicine, University of Freiburg, 79110 Freiburg im Breisgau, Germany; ramona.emig@tufts.edu (R.E.); remi.peyronnet@uniklinik-freiburg.de (R.P.); 4 CIBSS Centre for Integrative Biological Signalling Studies, Faculty of Biology, University of Freiburg, 79104 Freiburg im Breisgau, Germany; 5 Allergy Research Group, Department of Dermatology, Medical Center-University of Freiburg, Faculty of Medicine, University of Freiburg, 79106 Freiburg im Breisgau, Germany; philipp.esser@uniklinik-freiburg.de; 6 Division of Internal Medicine, University Hospital Basel, 4031 Basel, Switzerland

**Keywords:** BMP, BMP-2, BMP-4, TAA, VSMC, aortic aneurysm development

## Abstract

Vascular smooth muscle cells (VSMCs) play a key role in aortic aneurysm formation. Bone morphogenetic proteins (BMPs) have been implicated as important regulators of VSMC phenotype, and dysregulation of the BMP pathway has been shown to be associated with vascular diseases. The aim of this study was to investigate for the first time the effects of BMP-4 on the VSMC phenotype and to understand its role in the development of thoracic aortic aneurysms (TAAs). Using the angiotensin II (AngII) osmotic pump model in mice, aortas from mice with VSMC-specific BMP-4 deficiency showed changes similar to AngII-infused aortas, characterised by a loss of contractile markers, increased fibrosis, and activation of matrix metalloproteinase 9. When BMP-4 deficiency was combined with AngII infusion, there was a significantly higher rate of apoptosis and aortic dilatation. In vitro, VSMCs with mRNA silencing of BMP-4 displayed a dedifferentiated phenotype with activated canonical BMP signalling. In contrast, BMP-2-deficient VSMCs exhibited the opposite phenotype. The compensatory regulation between BMP-2 and BMP-4, with BMP-4 promoting the contractile phenotype, appeared to be independent of the canonical signalling pathway. Taken together, these results demonstrate the impact of VSMC-specific BMP-4 deficiency on TAA development.

## 1. Introduction

Dilatation of the aorta that exceeds the normal size of the aorta by more than 1.5 times is defined as aortic aneurysm [1], which is the second most common disease affecting the aorta [2]. Consequences of aortic complications are devastating, with mortality rates exceeding 94% [3]. Therefore, there is a great need for alternative treatments that delay, stop, or even reverse the pathology of aortic aneurysms [4]. Thoracic aortic aneurysms (TAAs) and abdominal aortic aneurysms share common pathophysiological features but differ in their aetiology and specific haemodynamic characteristics. TAAs show reduced invasion of myeloid cells, making them a good model to study the predominant role of vascular smooth muscle cells (VSMCs) in aneurysmal pathology [5].

During aneurysm development, the structural components of the thoracic aorta become dysregulated, resulting in vascular wall thinning, including progressive VSMC apoptosis and extracellular matrix (ECM) remodelling, which leads to a decrease in vascular elasticity and tension [6,7,8]. Together, this leads to a weakening of the arterial wall, TAA formation, and, if untreated, rupture of the aorta. VSMCs are the main cellular component of the aortic wall (35% of dry weight) and play a central role in maintaining aortic functions and homeostasis [9]. In contrast to their cardiac and skeletal muscle counterparts, VSMCs retain phenotypic plasticity and usually occur as a mixture of phenotypes [10]. In healthy blood vessels, the majority of VSMCs display a contractile (more differentiated) phenotype, characterised by an elongated, spindle-shaped morphology, expression of VSMC-specific contractile proteins (such as transgelin (TAGLN), smooth muscle actin (SMA), calponin (CALP), and myosin heavy chain 11 (MYH-11)), low production of ECM, and minimal proliferation and migration activity [11]. Through the integration of numerous environmental stimuli, such as changes in mechanical stimulation, contractile VSMCs can change their phenotype to become so-called synthetic VSMCs, a phenomenon known as phenotypic switching [7]. The synthetic phenotype is characterised by a less elongated, cobblestone morphology and a high rate of proliferation and migration, while markers of VSMC contractility are downregulated [12,13]. Interestingly, recent studies suggest that the VSMC phenotype switch occurs early in the development of TAAs, making it a promising therapeutic target [14]. 

The upstream signalling pathways that drive aneurysm development are not well understood. Recently, it has become evident that cytokine and growth factor signalling cascades such as the transforming growth factor-beta (TGF-β) pathway play a central role in the pathology of TAAs. Mutations in both TGF-β receptors 1 and 2 were associated with aneurysm development in Marfan syndrome type 2 and Loeys–Dietz syndrome [2]. Among the structurally conserved TGF-β family, bone morphogenetic proteins (BMPs) are the largest subfamily and are involved in the regulation of vascular development. BMPs signal through cell surface complexes of type I and type II serine/threonine kinase receptors [15]. All components of the BMP signalling pathway, ligands, receptors, and intracellular signalling molecules, are expressed in vascular cells [16]. Upon activation, the receptors mediate intracellular signalling via SMAD-1/-5/-9 transcription factors, which—when phosphorylated—modulate gene expression by interacting with SMAD-4. A well-known BMP signalling pathway target gene is the inhibitor of DNA binding 1 (ID-1) [17]. Beside this “canonical signalling pathway”, which is SMAD-dependent, there are several signalling pathways (acting, e.g., via MAPK) that are independent of SMAD and are summarised as “non-canonical signalling pathways” [18].

Recently, BMP family members have been demonstrated to be important regulators of VSMC plasticity. Several studies have reported their ability to promote VSMC differentiation into a contractile phenotype [19,20]. In contrast to TGF-β, very little is known about the role of the BMP signalling pathway in the pathogenesis of TAAs. BMP-2 and BMP-4 belong to the same BMP subgroup due to their receptor affinity and are known to be expressed by cardiomyocytes, endothelial cells, and VSMCs [20]. There is growing evidence that these two BMPs are important in vascular diseases such as pulmonary arterial hypertension or atherosclerosis [21,22]. The role of BMP-4 in TAAs, however, is poorly understood. Therefore, we now investigate the role of BMP-4 during the development of TAAs and the different effects of the highly homologous BMP-2 and BMP-4 in VSMCs.

## 2. Materials and Methods

### 2.1. Mice

Mice were housed under specific pathogen-free conditions and given ad libitum access to water and food. Animal handling and care was approved and in accordance with the guidelines for the care and use of laboratory animals published by Directive 2010/63/EU of the European Parliament. Animal protocols were approved in advance by the Regierungspräsidium Freiburg, Germany (approval reference G13-53 and G17-123). Tg(MYH-11-icre/ERT2)^1Soff^ (MYH-11Cre) mice were obtained from commercial sources with only male mice carrying the transgene on the Y-chromosome (please refer also to the major resources table). These mouse strains were crossed with B6.129BS-Bmp4^tm4Blh^ mice, a gift from Bridgid Hogan’s lab, to obtain MYH-11CreBMP-4flox (VSMCCtrl). Due to the heterozygous mating strategy, only male mice carry the CreERT2/BMP-4flox construct; this model allows for the study of male mice only. One advantage of using only male mice is that it limits the potential influence of female hormones, as many large population-based cohort studies have reported differences in atherosclerotic disease between the sexes [23]. BMP-4 deficiency in vascular smooth muscle cells was induced by intraperitoneal injection of 100 μL tamoxifen (TAM) (Sigma-Aldrich, Schnelldorf, Germany) (10 mg/mL) in corn oil daily for five days (Sigma-Aldrich, Schnelldorf, Germany) in VSMCCtrl mice to obtain VSMCΔBMP-4 mice. The tissue specificity of the TAM-induced deficiency in this mouse strain is well documented in the publication by Wirth et al. [24]. In 18–24-week-old VSMCCtrl and VSMCΔBMP-4, TAAs were induced 10 days after TAM induction by Angiotensin II (AngII) release from subcutaneous pumps for 28 days (see below) before the aortas were harvested for further analysis. For a more efficient cell culture, younger (four to six weeks old) VSMCCtrl and VSMCΔBMP-4 animals were used 10 days after the last injection to isolate VSMC. For long-term control of the model with CreERT2 and TAM, MYH-11Cre and VSMCCtrl mice were injected with TAM or corn oil as control for 10 days at 8–12 weeks of age and all 4 groups were examined after 4 months.

### 2.2. AngII Infusion Mouse Model for TAA

To induce TAA development, we used the AngII-infusion (Sigma-Aldrich, Schnelldorf, Germany) mouse model [25]. In this model, aortic aneurysm development is induced in the thoracic and abdominal aorta within 28 days of AngII infusion via subcutaneously implanted osmotic mini-pumps (ALZET^®^ Mini-Osmotic Pumps Model 2004; Durect Corporation, Cuptertino, CA, USA). Prior to osmotic pump implantation, mice were anaesthetised in an anaesthesia induction chamber (Tabletop Laboratory Animal Anaesthesia System, VetEquip Inc, Pleasanton, CA, USA), with 5% isoflurane (Abbott GmbH & Co. KG, Wiesbaden, Germany) mixed with 100% oxygen at a flow rate of 1 L/min, followed by a preemptive injection of caprofen (Rimadyl, Zoetis Deutschland GmbH, Berlin, Germany) (5 mg/kg bw, s.c.) for postoperative analgesia. Then, the mouse was placed in a supine position on an imaging platform. Throughout the examination, the mouse snout was kept in a nose cone connected to the anaesthesia system to maintain a steady-state sedation level (1–1.5% isoflurane mixed with 1 L/min 100% oxygen) while spontaneously breathing. Body temperature was monitored using a rectal thermometer (Indus Instruments, Houston, TX, USA) to continuously monitor and maintain the body temperature at 37 °C using a heated platform and a heat lamp. Dexpanthenol, an ophthalmic ointment (Bepanthen^®^ Bayer, Leverkusen, Germany), was applied on both eyes to prevent drying of the sclera. Using surgical tape, the four paws of the mouse were secured to the electrocardiogram electrodes of the imaging platform after applying electrode gel to monitor heart rate (HR). To remove chest fur, depilatory cream (Nair, Seattle, WA, USA) was applied to the chest from the sternum to the diaphragm using a cotton applicator tip. The cream was removed after 2 min with a gentle rolling motion of the tissue papers and then the chest was cleaned with aqua dest. moisturised gauze swab. Echocardiography and ultrasound examination of the aorta were performed. Osmotic pumps with 0.9% sodium chloride solution (natrium chloride, NaCl) as control or AngII (flow rate = 1 μg/kg/min) were implanted subcutaneously through a 1 cm incision in the neck perpendicular to the tail. A subcutaneous pocket was created in the back using blunt dissection, the pump was inserted, and the incision was closed with single-button sutures braided with 3-0 Mersilene^®^ (Ethicon, Norderstedt, Germany). The animals were placed in a red-light-warmed cage, observed until full consciousness was restored, and then returned to the colony. After 28 days, echocardiography and aortic ultrasound were performed again to assess the efficacy of AngII infusion in causing heart failure and aortic aneurysm development. The mice were then euthanised and the aortas isolated for further analysis. An investigation revealed that 6 out of 20 mice not only developed a TAA but also an abdominal aneurysm. Two of these animals died from abdominal aortic aneurysm rupture. 

### 2.3. Echocardiography

Echocardiography was performed by the high-resolution imaging system VEVO2100 system equipped with a 30 MHz, 100-frame-per-second micro-visualisation linier probe (MS400, mouse cardiovascular) (Visual Sonics, Toronto, ON, Canada). An acoustic coupling gel was applied over the chest wall of the anaesthetised and immobilised mouse to overcome air attenuation. Anaesthesia was maintained with 1.5–2.5% isoflurane throughout the echocardiographic examination to maintain the physiological HR. HRs were kept consistent between experimental groups (400–550 bpm). Cardiac image scanning was initiated with parasternal long- and short-axis views in B-mode and M-mode for assessment of left ventricular (LV) dimensions and systolic function. The M-mode imaging at the level of papillary muscles was used to acquire LV internal diameter (LVID), anterior wall thickness (LVAW), and posterior wall thickness (LVPW) at both the end systole and end diastole (LVIDs, LVAWs, LVPWs and LVIDd, LVAWd, LVPWd, respectively). In addition, LVID during systole and diastole was used to derive LV fractional shortening (FS) and the ejection fraction (EF). For assessment of the aortic diameters, the ascending aorta, transversal aorta, descending aorta, and abdominal aorta were measured using B-mode. All echocardiographic image (M-mode, 2D, and flows) measurements were performed, excluding the respiration peaks, and the average values were obtained from 5 or more cardiac cycles. All images were acquired within 10–25 min of anaesthesia induction, and calculations were done after acquisition.

### 2.4. Histological Examination and Immunostaining

Excised thoracic aorta descendens were embedded in Tissue-Tek O.C.T. compound (Fisher Scientific GmbH, Schwerte, Germany) and stored at −20 °C for further histological analysis. Five µm serial cryostat sections were cut starting from the thoracic aorta towards the abdominal aorta. The sections were air dried, fixed in ice-cold acetone, and subjected to standard Elastica van Gieson (EvG) staining (Morphisto GmbH, Frankfurt, Germany). Slides were analysed by a blinded investigator to assess morphological changes. The number of elastic lamella breaks and photographs were taken by Zeiss Axio Imager Z2 with ApoTome/ZEN 3.1 blue edition software. Immunofluorescence staining was performed for SMA FITC, MYH-11, TAGLN, collagen type III alpha 1 chain (COL3A1), MMP-9, and ID-1 compared to the respective immunoglobulin G (IgG) controls (please refer to the major resources table). AlexaFluor555-conjugated secondary antibodies were used and nuclei were stained with 4′,6-diamidino-2-phenylindol (DAPI) (Sigma-Aldrich, Schnelldorf, Germany). Using Fiji image analysis software (version 2.3.0) [26], an averaged threshold of the stack histogram of all images of a staining was calculated to determine the mean grey value of the aorta. For normalisation, the mean grey value of the inverse background was used.

Staining of human VSMC was performed with Phalloidin-TRITC (Sigma-Aldrich, Schnelldorf, Germany), αSMA-FITC, and TAGLN. AlexaFluor555-conjugated secondary antibodies were used and nuclei were stained with DAPI (Sigma-Aldrich, Schnelldorf, Germany). Photographs were taken by Zeiss Axio Imager Z2 with ApoTome/ZEN 3.1 blue-edition software.

### 2.5. Cell Culture

Human primary aortic VSMCs were purchased from Pelo Biotech (Martinsried, Germany) and cultured in smooth muscle cell growth medium (Pelo Biotech, Martinsried, Germany) supplemented with 5% foetal bovine serum (FBS, Thermo Fisher Scientific, Schwerte, Germany). Smooth muscle cells from fragmented mouse aortas were isolated by enzymatic digestion with collagenases (Roche Diagnostics, Mannheim, Germany) and cultured in DMEM (Thermo Fisher Scientific, Schwerte, Germany) with 15% FBS, strepomycin (Pelo Biotech, Planegg/Martinsried, Germany), and gentamicin (Pelo Biotech, Planegg/Martinsried, Germany) for, inter alia, nanoindentation experiments. 

Recombinant proteins were reconstituted according to the manufacturer’s protocol (R&D Systems, Wiesbaden, Germany). For recombinant protein or FBS stimulation, cells were seeded in culture medium and synchronised the next day in 0.4% FBS endothelial basal medium (EBM, Lonza, Verviers, Belgium) 24 h before stimulation. After initial serum starvation, VSMC were either stimulated with recombinant proteins every 24 h or incubated in 0.4% FBS/EBM as the control condition. For small interfering RNA (siRNA) transfection, cells were seeded the day before and transfected in EBM containing 1% FBS. 

### 2.6. Transfection

BMP-2 and/or BMP-4 deficiency in VSMCs was induced by siRNA transfection as recently described [27]. BMP-2 and BMP-4 siRNA were purchased from Thermo Fisher Scientific, Schwerte, Germany. Allstars negative control AlexaFluor488 was purchased from Qiagen, Hilden, Germany. For transfection, a final concentration of 100 nmol/L siRNA together with Lipofectamine RNAiMAX was used according to the manufacturer’s protocol (Thermo Fisher Scientific, Schwerte, Germany). Transfection efficacy was confirmed by quantitative real-time PCR (qRT-PCR) and Western blot analysis. 

### 2.7. Migration Assay

Cell migration assay was performed as previously described [28]. In brief, VSMCs transfected with siRNA for 72 h were labelled with 10 μM CFDA-SE (Thermo Fisher Scientific, Schwerte, Germany), harvested by centrifugation, resuspended in migration medium (EBM with 0.5% FBS, 0.1% BSA), counted, and placed in the upper chamber of a modified Boyden chamber (1 × 10^5^ cells per HTS FluoroBlok 24-well chamber; pore size 8 μm; BD Biosciences, Heidelberg, Germany). The chambers were placed in 24-well culture dishes containing migration medium and indicated recombinant proteins. After incubation for 4 h at 37 °C, 5% CO_2_ the cells were fixed with 4% PFA and migrated cells were counted manually in 5 random microscopic fields using an Axiovert fluorescence microscope. A positive control was performed with 40 ng/mL platelet-derived growth factor (PDGF-BB) (R&D Systems, Wiesbaden, Germany).

### 2.8. Collagen–Gel Contraction Assay

Twenty-four hours after siRNA transfection, 5 × 10^4^ VSMC were mixed with 250 µL freshly prepared collagen gel solution (Cultrex Rat Collagen I (R&D Systems, Wiesbaden, Germany), 0.1% acetic acid, 1 M sodium hydroxide solution, 1 M HEPES buffer (Thermo Fisher Scientific, Schwerte, Germany), 10× Medium 199 (Sigma-Aldrich, Schnelldorf, Germany)); seeded in 48 well plates; and incubated for 30 min at 37 °C to induce gelation. Afterwards, 250 µL 0.4% FBS/EBM were added for 48 h before the gels were lifted off the bottom of the wells and allowed to float freely. Twenty-four hours later, the gels were imaged and the gel size was quantified with a digital imaging system (ChemiDOC XRS (Bio-Rad, Munich, Germany)). A control for the inhibition of gel contraction was performed with 1 M butanedione monoxime (BDM, Sigma-Aldrich, Schnelldorf, Germany). 

### 2.9. Proximity Ligation Assay (PLA)

To detect direct BMP receptor 1a (BMPR1a) phosphorylation, VSMCs were grown on 12-well glass slide covers (ibidi, Graefelfing, Germany), fixed with acetone for 20 min at −20 °C, and washed 3 times in phosphate-buffered saline. Subsequently, the Duolink^®^ PLA assay was performed following the manufacturer’s instructions (Sigma-Aldrich, Schnelldorf, Germany). In brief, anti-BMPR1a antibody and phosphoserin/phosphothreonin antibody were prepared following the Duolink^®^ PLA probemaker protocol. Afterwards the antibodies were incubated overnight on the slides at 4 °C followed by a ligase and polymerase reaction to amplify the signal. DAPI was used to stain the nuclei before photographs were taken by Zeiss Axio Imager Z2 with ApoTome/ZEN 3.1 blue edition software. Using Fiji image analysis software (version 2.3.0) [26], a threshold was defined to count all fluorescent dots in the PLA stain and relate them to the number of DAPI-detected nuclei. The relative data compared to the control condition are shown.

### 2.10. In Situ Zymography on Slides (MMP Activity)

To analyse the activity of the MMP present in the tissue, especially the gelatinases MMP-2 and MMP-9 [29,30], DQ gelatine from the EnzCheck© kit from Thermo Fisher Scientific (Molecular Probes) was used. A reaction buffer of 0.2 mM sodium azide, 150 mM NaCl, 5 mM CaCl_2_, and 50 mM TrisHCl was used. The slides were diluted in a solution of 1 mg/mL DQ gelatine solution in a 1:50 ratio with the buffer. After washing with aqua dest., the sections were fixed in 4% formalin. As a negative control to inhibit the activity of the metalloproteinases, a slide was pre-incubated with 20 mM ethylenediaminetetraacetic acid (EDTA) for one hour before starting the experiment.

### 2.11. TUNEL (TdT-Mediated dUTP-Biotin Nick End Labelling) Staining

To detect apoptotic nuclei, the “In Situ Cell Death Detection Kit” of Roche Applied Sciences (Basel, Switzerland) was used according to the manufacturer’s protocol. The labelling solution supplied in the kit was used as a negative control without prior addition of the enzyme solution.

### 2.12. Nanoindentation

To characterise the mechanical properties of cultured VSMCs, nanoindentation experiments were performed as previously described for human atrial fibroblasts [31]. In brief, human or murine VSMCs were seeded at a density of 3–4 × 10^4^ cells and transfected. Mechanical testing was performed using the Chiaro nanoindenter (Optics11, Amsterdam, The Netherlands) 3 days after knockdown or knockout induction, respectively. A spherical glass tip (3.0–3.4 µm radius) attached to a pre-calibrated cantilever with a spring constant between 0.012 and 0.015 N/m (Optics11) was used to indent the cells. Each indentation was started 4–6 µm above the cell surface and consisted of 3 distinct phases: First, a displacement of 10 µm was initiated with a speed of 5 µm/s. Then, the probe was held at maximal motor displacement for 2 s before finally being retracted to the starting position at the same speed. For each cell, 3 measurements at 3 different positions were performed. The effective Young’s modulus (E_eff_) was derived from force vs. indentation curves using the Hertzian model for contact mechanics under the assumption of a Poisson ratio of 0.5, which is customarily used for mechanical testing of biological materials (Data-Viewer2.3, Optics11). As a measure of cell viscosity, the dominant time constant of stress relaxation during the second phase of the indentation protocol was calculated using an in-house Matlab script (Version: 9.6.0.1214997 (R2019a) Update 6, MatWorks Inc., Natick, MA, USA). Data are presented using Violin Superplots [32]. Violin SuperPlots include information on data heterogeneity: The normalised density estimates of individual replicates are stacked to show how each replicate (color-coded stripe) contributes to the overall density estimate (outline). 

### 2.13. RNA Extraction and Reverse Transcription

Total RNA from mouse aorta was extracted using the TriPure isolation method followed by DNase I treatment according to the manufacturer’s protocol (Sigma-Aldrich, Schnelldorf, Germany). To extract RNA from cultured VSMCs, the Aurum Total RNA Mini Kit (Bio-Rad) was used following the manufacturer’s protocol. Reverse transcription was performed with iScript cDNA-Kit, applying 1 µg RNA following the manufacturer’s protocol (Bio-Rad, Munich, Germany).

### 2.14. Quantitative Real-Time PCR

qRT-PCR was performed using IQ SybrGreen 2× Supermix (BioRad, Munich, Germany) and was analysed with the CFX96 touch real-time PCR detection system (Bio-Rad, Munich, Germany). Quantification was performed using CFX manager version 3.1 software (Bio-Rad, Munich, Germany). Differences in gene expression were calculated using the ΔΔCT method [33]. The housekeeping gene human RNA polymerase II (hRP) and m36B4 for murine samples were used for internal normalisation. Primers were purchased from Eurofins MWG Operon, Ebersberg, Germany. For primer sequences please refer to the major resources table.

### 2.15. Protein Extraction

To extract proteins from the mouse aortas, 100 μL RIPA buffer (RIPA + protease inhibitor (1:200) + phosphatase inhibitor (1:50)) were added to the aorta and homogenised with an electric homogeniser. After incubation for 20 min at 4 °C, lysates were centrifuged at 10,000× *g*, 4 °C for 10 min. The supernatant was used for protein analysis. Proteins from cultured VSMC were extracted by incubating the cells for 10 to 20 min with a solution of Tris-HCl, EDTA, NaCl, Igepal 10%, sodium deoxycholate 20%, SDS 20%, and proteinase inhibitor diluted 1:200 in this solution. The mass was homogenised by using a cell scraper. Afterwards, centrifugation was performed at 10,000× *g*, 4 °C for 10 min. The supernatant was used for protein analysis by Western blot analysis.

### 2.16. Western Blot Analysis

Western blot analysis was performed as previously described [28]. Primary antibodies were incubated overnight at 4 °C and secondary antibodies at room temperature for 1 h in 3% non-fat dried milk/TBST. Visualisation was performed by an ECL system (GE Healthcare Europe GmbH, Freiburg, Germany) and a digital imaging system (ChemiDOC XRS (Bio-Rad, Munich, Germany). For quantification of protein band intensities, Image Lab (Bio-Rad, Munich, Germany) was used and expression was normalised to GAPDH loading control. The full unedited Western blots are shown in Figure S11.

### 2.17. Statistical Analysis

Statistical analysis was performed using GraphPad Prism 9, La Jolla, CA, USA. Data are presented as mean ± SD. Comparisons between two groups were made by Student’s *t*-test (2-sided, unpaired); comparisons between multiple groups were made by ordinary one-way ANOVA-test with Bonferroni correction for multiple testing. All experiments were repeated at least three times in triplicate. Results were considered statistically significant for *p* < 0.05.

## 3. Results

### 3.1. BMP-4 Stimulation Results in Activation of Canonical BMP Signalling In Vitro

**Figure 1:**
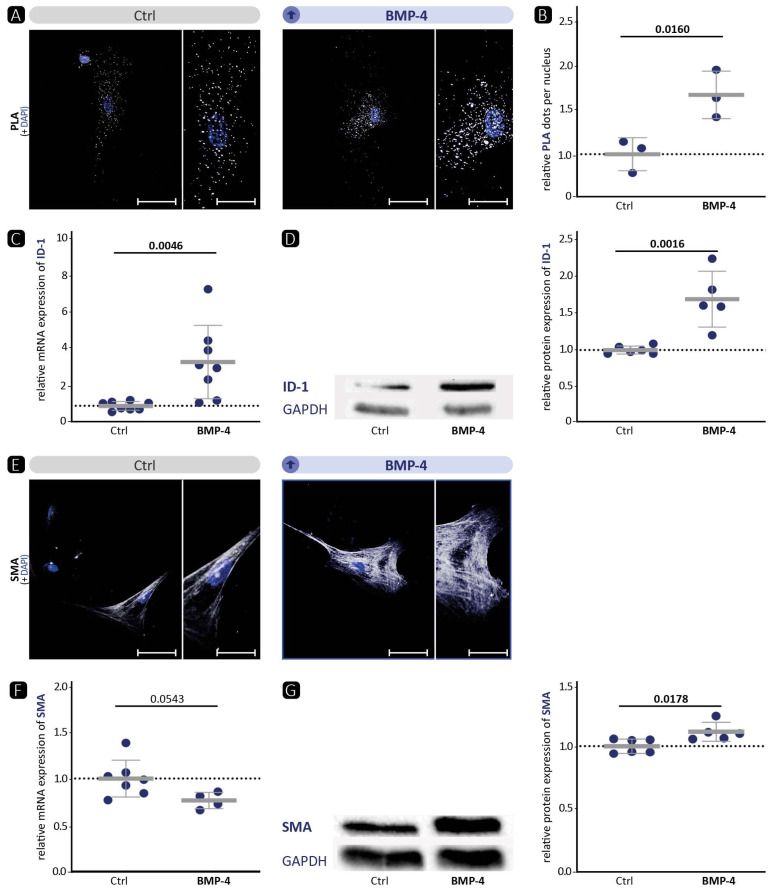
BMP-4 activates the canonical BMP signalling pathway, elevates expression of the target protein ID-1, and promotes the contractile phenotype of human VSMCs in vitro. VSMCs were either incubated in 0.4% FBS/EBM (Ctrl) or stimulated with BMP-4 (40 ng/mL). Experiments were performed after 72 h of BMP-4 stimulation. Data are presented as mean ± SD and comparisons were calculated by an unpaired Student’s *t*-test; *p*-values as indicated. (**A**,**E**) Representative photomicrographs of (**A**) in-situ PLA for the detection of BMPR1a phosphorylation after additional stimulation with BMP-4 10 min before analysis and (**E**) immunostaining against SMA. Nuclei were stained with DAPI (blue). Left image: scale bar 50 µm; right image: zoomed in to 200%, scale bar 25 µm. (**B**) Relative quantification of PLA-positive dots per DAPI-detected nucleus (*n ≥* 3). (**C**,**F**) qRT-PCR of (**C**) ID-1 (*n ≥* 8) and (**F**) SMA (*n ≥* 4). (**D**,**G**) Western blot analysis of (**D**) ID-1 (*n ≥* 6) and (**G**) SMA (*n ≥* 5) with GAPDH as loading control.

The aim of this study was to understand the role of BMP-4 on VSMC phenotypes and its impact on TAA genesis. Therefore, we first investigated the effect of BMP-4 treatment on human aortic VSMCs to check whether the BMP signalling pathway can be activated. In all cell culture experiments, the 72 h time point after treatment is shown unless indicated otherwise, as the effects were most evident at this time point (48 h BMP-4 treatment is shown in Supplementary Figure S1). The proximity ligation assay (PLA) revealed that stimulation of VSMCs with BMP-4 in vitro resulted in higher phosphorylation of BMP receptor BMPR-1a as one of the first steps of BMP signalling pathway activation (Figure 1A,B). Consequently, the expression of the downstream canonical BMP pathway involving the transcription factor ID-1 was increased in response to stimulation with BMP-4 at the mRNA and protein level (Figure 1C,D). More prominent contractile fibres were detected by immunofluorescence staining of SMA (Figure 1E) and TAGLN (Figure S1D) as a sign of a more pronounced contractile phenotype. This was supported by a higher protein expression of TAGLN (Figure S1J) and SMA (Figure 1G), as well as mRNA expression of CALP (Figure S1K). Taken together, we show that BMP-4 stimulation of human aortic VSMCs in vitro leads to an activation of canonical BMP signalling and subsequently higher expression of contractile markers.

### 3.2. Impact of VSMC-Specific BMP-4-Deficiency on TAA Development In Vivo

To further investigate the role of BMP-4 in the aortic wall, we then assessed the effect of BMP-4 deficiency compared with the manifestation of AngII-induced TAAs and the simultaneous treatment with AngII and BMP-4 deficiency in VSMCΔBMP-4 mice. In-vivo VSMC-specific BMP-4 deficiency (VSMCΔBMP-4) was induced by tamoxifen injection in VSMCCtrl. There is growing evidence that the Cre-LoxP system can have toxic effects on cells independent of its ability to recombine pairs of artificial LoxP sites in target genes [34]. Mice expressing CreERT2 but lacking the floxed target genes (MYH-11Cre) with (+TAM) and without tamoxifen (∅ TAM) treatment were compared with MYH-11CreBMP-4flox mice with (VSMCΔBMP-4) and without (VSMCCtrl) tamoxifen treatment to assess possible effects due to tamoxifen toxicity and CreERT2 activation. As shown in Supplementary Figure S2A, no changes in wall thickness or elastic fibre ruptures in aortas from TAM treated or untreated BMP-4-competent mice (MYH-11Cre ∅ TAM, MYH-11Cre + TAM, VSMCCtrl) were observed. Only VSMCΔBMP-4 mice showed larger minimal and mean wall thickness as well as more elastic fibre ruptures in contrast to VSMCCtrl animals (Figure S2A). Accordingly, analysis of immunohistochemistry (IHC) showed that expression of the contractile markers SMA and TAGLN was not significantly altered in BMP-4 competent mice with or without TAM treatment, whereas TAM treatment in MYH11CreBMP-4flox mice (VSMCΔBMP-4) led to significant changes due to BMP-4 abrogation in VSMC (Figure S2B,C).

**Figure 2:**
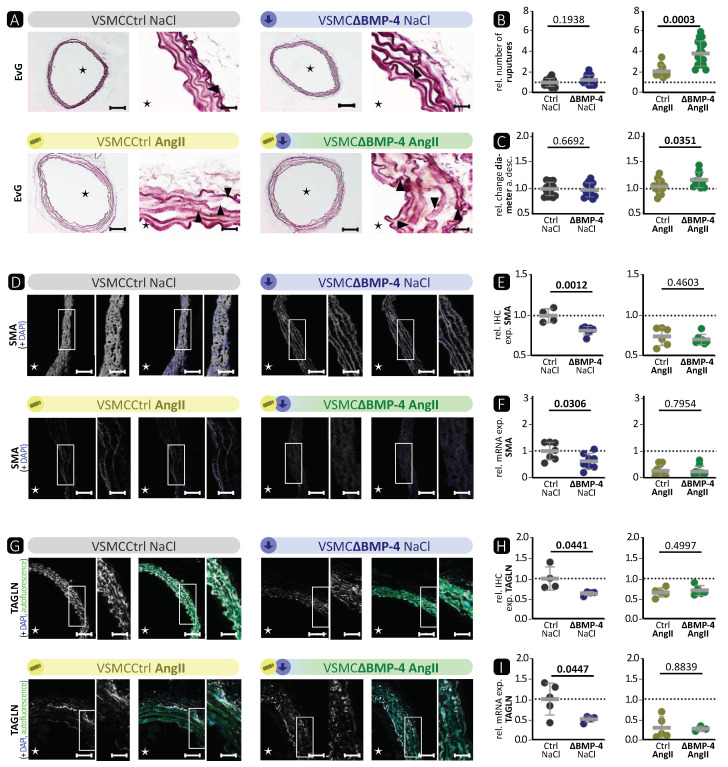
BMP-4 deficiency in murine VSMCs reduces expression of contractile markers and enhances AngII-driven destruction of the thoracic aortic wall. A subcutaneous pump with AngII (flow rate = 1 µg/kg/min) or 0.9% NaCl was implanted in phenotypic wild-type littermates VSMCCtrl (Ctrl) or VSMCΔBMP-4 mice (ΔBMP-4) 28 days before the thoracic aorta was isolated. Data are presented as mean ± SD and comparisons were calculated by an unpaired Student’s *t*-test; *p*-values as indicated. Rel. = relative; exp. = expression. (**A**) EvG. Ruptures of elastic fibres are marked with triangles, vascular lumen is marked with an asterisk Scale bars: left side 200 µm, right side: 20 µm. (**B**) Quantification of ruptures of elastic fibres (*n ≥* 10). (**C**) Relative change over 28 days in aortic diameter of the aorta descendens (a. desc.) (*n ≥* 10). (**D**,**G**) Representative photomicrographs of immunostaining against (**D**) SMA and (**G**) TAGLN. Vascular lumen is marked with an asterisk; left image: scale bar 100 µm; right image: zoomed to 200% of the highlighted area, scale bar 50 µm. DAPI stain visualises nuclei (blue). Autofluorescence of elastic fibres in green when indicated. (**E**,**H**) Mean density of IHC of (**E**) SMA (*n ≥* 4) and (**H**) TAGLN (*n ≥* 3). (**F**,**I**) qRT-PCR analysis of (**F**) SMA (*n ≥* 7) and (**I**) TAGLN (*n ≥* 3).

As we identified the activation of the canonical signalling pathway and induction of the contractile phenotype in VSMC after BMP-4 treatment in vitro, we next addressed the impact of BMP-4 deficiency on TAA development induced by AngII infusion. Therefore, we first checked the efficiency of BMP-4 deficiency in VSMCs on mRNA and protein levels following TAM injection (Figure S3A,B) in mice with implanted subcutaneous pumps releasing either NaCl as the control or AngII as a TAA inducer. Consistent with the reduced expression of BMP-4, reduced expression of ID-1 as the target gene of the canonical BMP pathway was detected by immunohistology only in VSMCΔBMP-4 mice (Figure S3C,D). BMP-4 deficiency itself did not significantly affect the number of elastic fibre ruptures in the vessel wall, whereas combining BMP-4 deficiency and AngII induction of TAA led to an increased effect size, with significantly more elastic fibre ruptures (Figure 2A,B) and a thickening (maximal and mean thickness) of the vessel wall (Figure S3F), resulting in an even greater dilatation of the descending aorta (Figure 2C) compared to AngII treatment alone. VSMC-specific BMP-4 deficiency itself was sufficient to cause significantly lower mRNA and IHC expression of the three contractile markers SMA (Figure 2D–F) and TAGLN (Figure 2G,H,I), as well as IHC expression of MYH-11 (Figure S3H,I) in thoracic aortas, leading to an comparable effect, as observed after AngII treatment. In addition, a reduced level of contractile marker expression as result of the deficiency of BMP-4 was confirmed by mRNA analysis of isolated VSMCs from VSMCCtrl and VSMCΔBMP-4 mice (Figure S4A–D). To further characterise passive mechanical properties of these isolated murine VSMCs, we performed nanoindentation experiments. Consistent with the reduction in contractile marker expression, VSMCs isolated from VSMCΔBMP-4 mice were significantly softer and relaxed faster compared to those isolated from VSMCCtrl (Figure S4E). Interestingly, combinatory treatment of BMP-4-deficient mice with AngII did not result in further reduction regarding the downregulation of SMA, TAGLN, or MYH-11 expression (Figure 2D–I and Figure S3H–J) in contrast to the additive effects on fibre rupture, vessel wall thickening, and aorta dilatation, indicating that BMP-4 deficiency in VSMCs is already sufficient to induce a pre-stage of a TAA phenotype.

**Figure 3:**
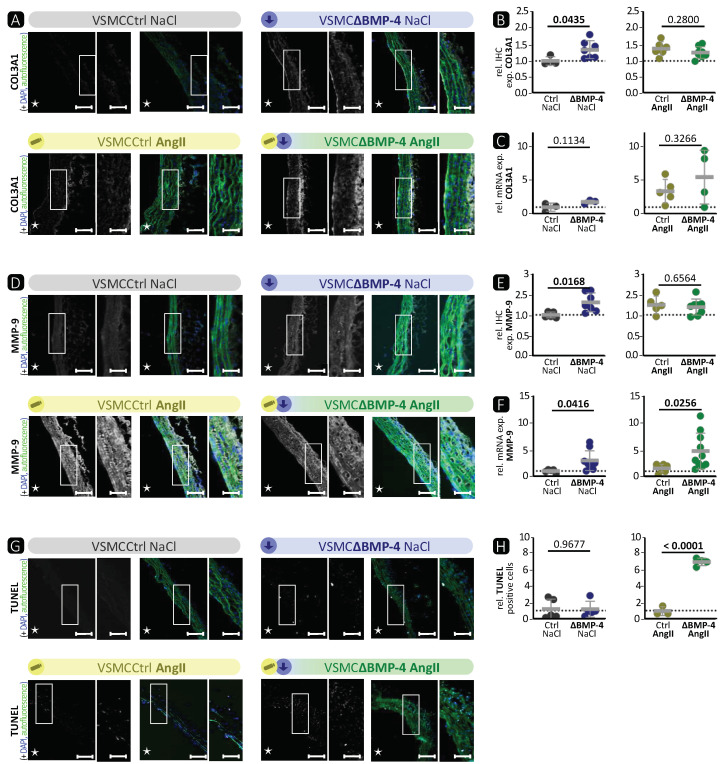
BMP-4 deficiency in murine VSMCs causes an elevation of pathological ECM remodelling characteristic in the thoracic aorta. A subcutaneous pump with AngII (flow rate = 1 µg/kg/min) or 0.9% NaCl was implanted in phenotypic wild-type littermates VSMCCtrl (Ctrl) or VSMCΔBMP-4 mice (ΔBMP-4) 28 days before the thoracic aorta was isolated. Data are presented as mean ± SD and comparisons were calculated by an unpaired Student’s *t*-test; *p*-values as indicated. Rel. = relative; exp. = expression. (**A**,**D**) Representative photomicrographs of immunostaining against (**A**) COL3A1 and (**D**) MMP-9. Vascular lumen is marked with an asterisk. Left image: scale bar 100 µm; right image: zoomed to 200% of the highlighted area, scale bar 50 µm. DAPI stain visualises nuclei (blue). Autofluorescence of elastic fibres in green. (**B**,**E**) Mean density of the IHC of (**B**) COL3A1 (*n ≥* 3) and (**E**) MMP-9 (*n ≥* 5). (**C**,**F**) qRT-PCR analysis of (**C**) COL3A1 (*n ≥* 3) and (**F**) MMP-9 (*n ≥* 4). (**G**) Representative photomicrographs of TUNEL assay. Vascular lumen is marked with an asterisk. Left image: scale bar 100 µm; right image: zoomed to 200% of the highlighted area, scale bar 50 µm. DAPI stain visualises nuclei. (**H**) Quantification of TUNEL positive cells (*n ≥* 3).

Next, we decided to address the effect of BMP-4 deficiency on ECM remodelling. IHC of COL3A1 revealed a significantly elevated formation of collagen fibres in VSMCΔBMP-4 mice compared to VSMCCtrl, which was similar to the effect of AngII (Figure 3A,B). However, there was no significant effect observed on the mRNA level (Figure 3C), which may reflect post-translational regulatory mechanisms that affect protein levels independently of mRNA transcription. As expected, IHC and Western blot analysis showed higher expression of MMP-9 (Figure 3D,E and Figure S5A) and activity of MMPs by BMP-4 deficiency (Figure S5B). At the mRNA level, there was even an additive effect with a significant increase in MMP-9 expression in VSMCΔBMP-4 with AngII compared to VSMCCtrl with AngII (Figure 3F). Only in VSMCΔBMP-4 mice with simultaneous AngII infusion was a significantly higher number of apoptotic cells observed (Figure 3G,H). 

Taken together, these results show that VSMCΔBMP-4 leads to a downregulation of the BMP signalling pathway target ID-1 and a lower contractile phenotype of VSMCs. As anticipated, the opposite effect to BMP-4 stimulation in VSMCs in vitro was observed (Figure 1). In vivo, we observed in VSMCΔBMP-4 mice a higher destruction of the ECM, which in combination with AngII leads to higher apoptosis, a widened aortic wall, and more extensive TAA formation. 

To study the effect of BMP-4 deficiency on cardiac function with and without AngII influence, echocardiography was performed on days 0 and 28 of pump implantation. An AngII effect on LVAWs was only seen in VSMCΔBMP-4 mice and not in VSMCCtrl mice (Figure S6A). Additive effects were observed in EF and FS. Both BMP-4 deficiency and AngII infusion separately had minimal effects, while the combination resulted in a significant reduction in EF and FS compared to controls (Figure S6D).

Taken together, it can be concluded that VSMCΔBMP-4 mice show comparable effects to mice after AngII infusion. AngII application to VSMCΔBMP-4 promotes aortic aneurysm development and leads to cardiac stress.

### 3.3. BMP-4 Deficiency Affects the Canonical BMP Pathway and VSMC Phenotype

**Figure 4:**
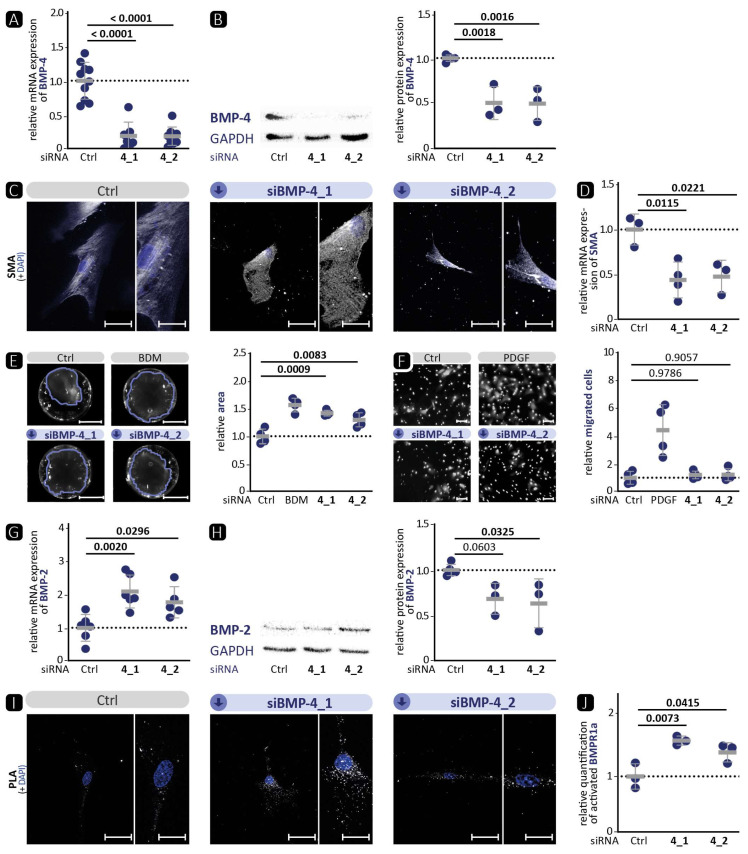
BMP-4 deficiency in human aortic VSMCs affects the contractile phenotype, elevates BMP-2 expression, and induces BMP signalling pathway activation. BMP-4 was silenced in VSMCs by either of two specific siRNA targeted against BMP-4 (4_1, 4_2). Scrambled siRNA was used as a control (Ctrl). Experiments were performed 72 h post siRNA transfection. Data are presented as mean ± SD and comparisons were calculated by an ordinary one-way ANOVA test with Bonferroni correction for multiple testing; *p*-values as indicated. (**A**,**D**,**G**) qRT-PCR of (**A**) BMP-4 (*n ≥* 7), (**D**) SMA (*n ≥* 3), and (**G**) BMP-2 (*n ≥* 5). (**B**,**H**) Western blot analysis of (**B**) BMP-4 (*n ≥* 3) and (**H**) BMP-2 (*n ≥* 3) with GAPDH as loading control. (**C**) Representative photomicrographs of immunostaining against SMA. DAPI staining visualises nuclei (blue). Left image: scale bar 50 µm, right image: zoomed in to 200%, scale bar 25 µm. (**E**) Representative images with marked collagen area and quantification of collagen gel contraction assay (*n ≥* 3). As a positive control, the contractility was inhibited using BDM (1 M). Scale bar 5 mm. (**F**) Representative photomicrographs and quantification of transmigration assay with PDGF (40 ng/mL) as positive control for higher VSMC migration. Scale bar 50 µm. (*n ≥* 3). (**I**) Representative photomicrographs of in-situ PLA for the detection of BMPR1a phosphorylation. DAPI staining visualises nuclei (blue). Left image: scale bar 50 µm, right image: zoomed in to 200%, scale bar 25 µm. (**J**) Relative quantification of PLA-positive dots per DAPI-detected nucleus (*n ≥* 3).

To further analyse the effect of BMP-4-deficiency on the cellular level, validate our previous data from the TAM model (Figure 2), and test for transferability of the results to the human system, we investigated the effect of silencing BMP-4 by siRNA in human aortic VSMCs (Figure 4). Seventy-two hours post BMP-4-specific siRNA transfection, mRNA and protein expression were analysed. The results of the 48 h time point can be found in the Supplementary Materials (Figure S7). Using two different siRNA (siBMP-4_1 and siBMP-4_2), we detected significantly reduced BMP-4 expression at the mRNA and protein levels (Figure 4A,B). In line with the results of BMP-4 deficiency in murine aortic tissue (Figure 2), deficiency of BMP-4 induced by either of the two siRNA resulted in significantly lower mRNA expression of the contractile markers SMA (Figure 4D) as well as CALP (Figure S7D) by the first sequence compared to control cells transfected with non-targeting control siRNA. In contrast, Western blot showed stronger protein expression of SMA (Figure S7G) and TAGLN (Figure S7K). However, immunocytochemical staining of SMA (Figure 4C) and TAGLN (Figure S7L) showed fewer contractile fibres, which resembles the migratory, synthetic phenotype. Accordingly, the ability of the cells to contract collagen in vitro was significantly lower after the silencing of BMP-4 (Figure 4E). In contrast, there was no significant effect of BMP-4 silencing detected on the migration of VSMCs (Figure 4F). 

Due to these inconsistent results that did not clearly support a synthetic phenotype, we analysed the second BMP of the subgroup, the highly homologous BMP-2 [35], as it is known that autoregulation takes place in the BMP signalling pathway [36]. Interestingly, we found that BMP-2 expression was increased upon BMP-4 deficiency at the mRNA level, whereas it tended to be lower on the protein level (Figure 4G,H). As we used cell lysates for Western blot analysis, this reversed effect may be explained by the high homology of BMP-2 and BMP-4 resulting in unspecific binding of the BMP-2 antibody to BMP-4 [37]. Finally, using PLA, a higher phosphorylation of BMPR1a was detected in response to BMP-4 silencing (Figure 4I,J), although the opposite would have been expected from the stimulation experiments (Figure 1). 

Taken together, silencing of BMP-4 in human aortic VSMCs reduced contractile fibre formation and collagen contraction similar to in vivo experiments (Figure 2), but in contrast to in vivo experiments, activation of the canonical BMP signalling pathway was observed, possibly due to the concomitant upregulation of the BMP-4 homolog BMP-2.

### 3.4. Effect of BMP-2 on the Canonical BMP Signalling Pathway and the Contractile Phenotype of VSMCs

**Figure 5:**
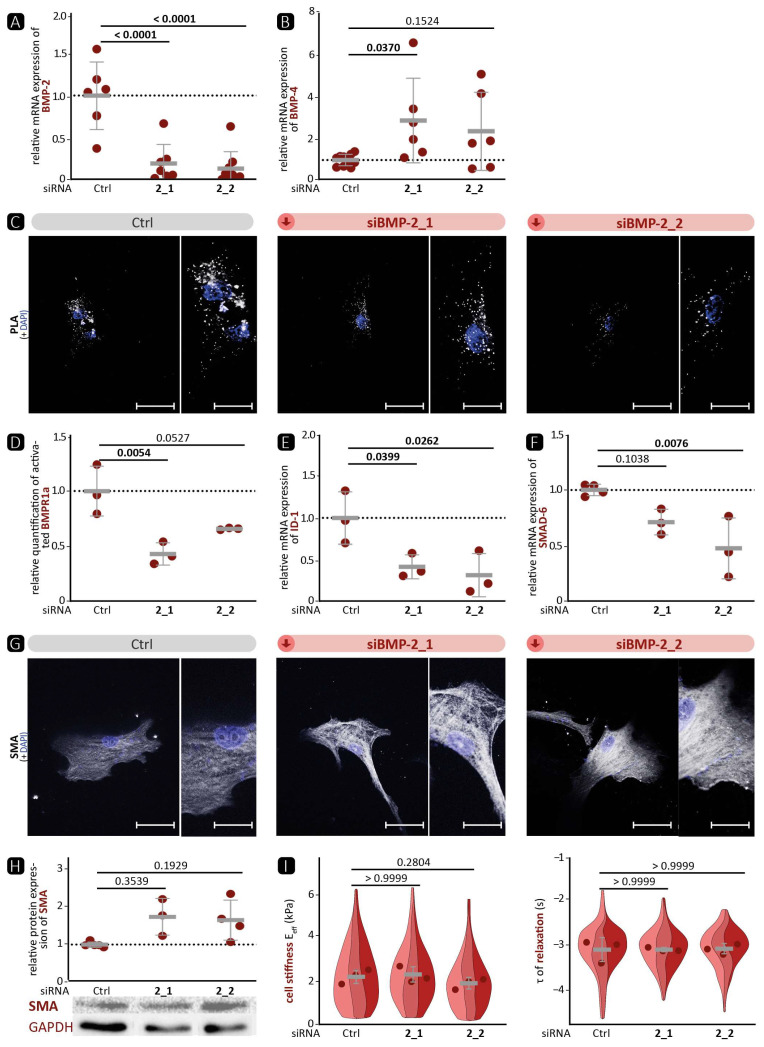
BMP-2 deficiency leads to stronger expression of BMP-4 but reduces activation of the signalling pathway and promotes a contractile phenotype. BMP-2 was silenced in human VSMC by either of two specific siRNAs targeted against BMP-2 (2_1, 2_2). Scrambled siRNA was used as control (Ctrl). Experiments were performed 72 h post siRNA transfection unless indicated otherwise. Data are presented as mean ± SD, and comparisons were calculated by an ordinary one-way ANOVA-test with Bonferroni correction for multiple testing; *p*-values as indicated. Rel. = relative; exp. = expression. (**A**,**B**,**E**,**F**) qRT-PCR of (**A**) BMP-2 (*n ≥* 6), (**B**) BMP-4 (*n ≥* 6), (**E**) ID-1 (*n ≥* 3), and (**F**) SMAD-6 (*n ≥* 3). (**C**,**G**) Representative photomicrographs of (**C**) in-situ PLA for the detection of BMPR1a phosphorylation and (**G**) of immunostaining against SMA. DAPI stain visualises nuclei (blue). Left image: scale bar 50 µm; right image: zoomed in to 200%, scale bar 25 µm. (**D**) Relative quantification of PLA-positive dots per DAPI-detected nucleus (*n ≥* 3). (**H**) Western blot analysis of SMA with GAPDH as loading control (*n ≥* 3). (**I**) Nanoindentation: effective Young’s modulus E_eff_ and time constant τ of relaxation 48 h post siRNA transfection (3 experiments, each *n ≥* 13).

To further investigate the differences between BMP-2 and BMP-4 on the phenotype of VSMC, BMP-2 was silenced for 72 h using BMP-2-specific siRNA in human aortic VSMCs. Data 48 h post transfection are shown in the supplement (Figure S8). As for BMP-4 deficiency (Figure 4), siRNA-induced BMP-2 deficiency increased BMP-4 mRNA expression (Figure 5A,B), whereas protein expression was not significantly affected (Figure S8G). We hypothesise that the apparent increase in BMP-2 expression observed by Western blotting (Figure S8B,D) was again due to the elevated expression of BMP-4. As expected, reduced BMP-2 expression resulted in diminished phosphorylation of the BMPR1 receptor, as shown by the PLA assay (Figure 5C,D). Consequently, ID-1 and SMAD-6 expression was reduced in response to BMP-2 silencing (Figure 5E,F). In parallel to the stronger expression of BMP-4, analysis of the contractile VSMC markers after BMP-2 silencing revealed a tendency towards elevated protein expression of SMA (Figure 5H) and TAGLN (Figure S8T) and stronger contractile fibre formation (Figure 5G and Figure S8P). However, nanoindentation experiments 48 h post transfection showed no significant differences in cell stiffness or time course of relaxation between silenced and control BMP-2 cells (Figure 5I). 

Together, silencing of BMP-2 led to reduced canonical BMP pathway signalling accompanied by a compensatory BMP-4 upregulation on the mRNA level with higher contractile marker expression but no significant changes in VSMC mechanical properties.

### 3.5. Effect of Simultaneous BMP-2/-4 Deficiency as Well as of the Use of BMP Inhibitors

**Figure 6:**
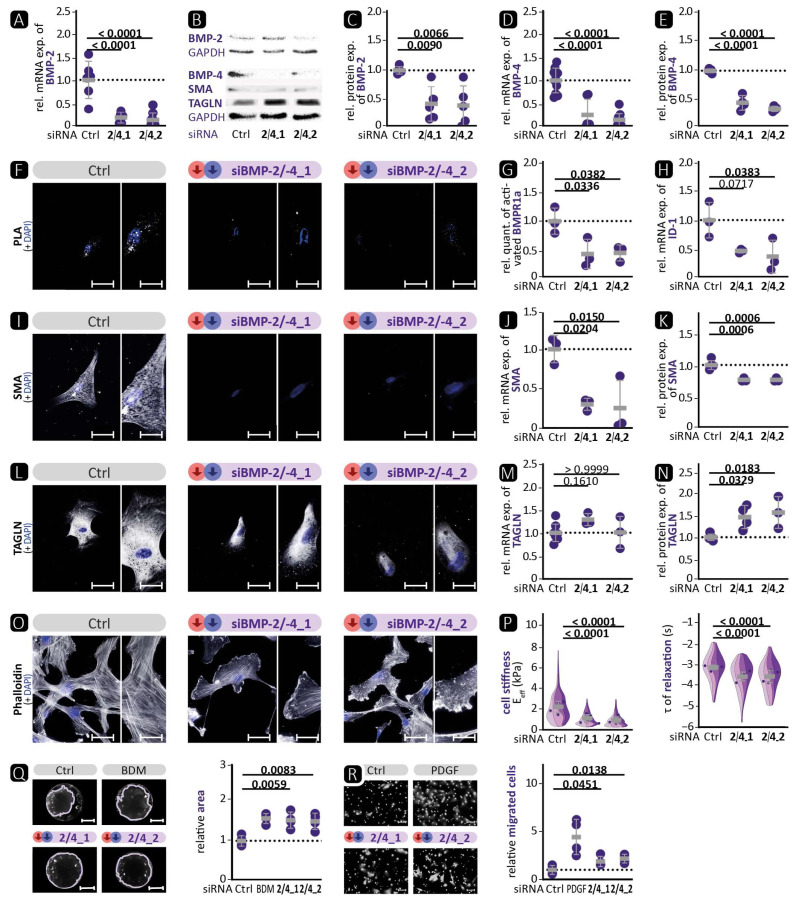
Simultaneous deficiency of BMP-2/-4 led to reduced BMP pathway activation and loss of the contractile VSMC phenotype. Human VSMCs were transfected with a combination of BMP-2 and BMP-4 targeting siRNA (2/4_1, 2/4_2) compared to scrambled siRNA (Ctrl) as control. Experiments were performed 72 h post treatment or as indicated otherwise. Data are presented as mean ± SD and comparisons were calculated by an ordinary one-way ANOVA-test with Bonferroni correction for multiple testing; *p*-values as indicated. Rel. = relative; exp. = expression. (**A**,**D**,**H**,**J**,**M**) qRT-PCR of (**A**) BMP-2 (*n ≥* 4), (**D**) BMP-4 (*n ≥* 4), (**H**) ID-1 (*n ≥* 3), (**J**) SMA (*n ≥* 3), and (**M**) TAGLN (*n ≥* 3). (**B**,**C**,**E**,**K**,**N**) Western blot analysis of (**C**) BMP-2 (*n ≥* 5), (**E**) BMP-4 (*n ≥* 4), (**K**) SMA (*n ≥* 3), and (**N**) TAGLN (*n ≥* 3) with GAPDH as loading control; representative blots shown in (**B**). (**F**,**I**,**L**,**O**) Representative photomicrographs of (**F**) in-situ proximity ligation assay (PLA) for the detection of BMPR1a phosphorylation and immunostaining of (**I**) SMA, (**L**) TAGLN, and (**O**) phalloidin. DAPI stain visualises nuclei (blue). Left image: scale bar 50 µm; right image: zoomed in to 200%, scale bar 25 µm. (**G**) Relative quantification of PLA-positive dots per DAPI-detected nucleus (*n ≥* 3). (**P**) Nanoindentation: effective Young’s modulus E_eff_ and time constant τ of relaxation 48 h post siRNA transfection (4 experiments, each *n ≥* 13). (**Q**) Representative images with marked collagen area and quantification of collagen gel contraction assay (*n ≥* 3). As a positive control, the contractility was inhibited using BDM (1 M). Scale bar 5 mm (**R**) Representative photomicrographs and quantification of transmigration assay with PDGF (40 ng/mL) as positive control for higher VSMC migration. Scale bar 50 µm. (*n ≥* 3).

**Figure 7:**
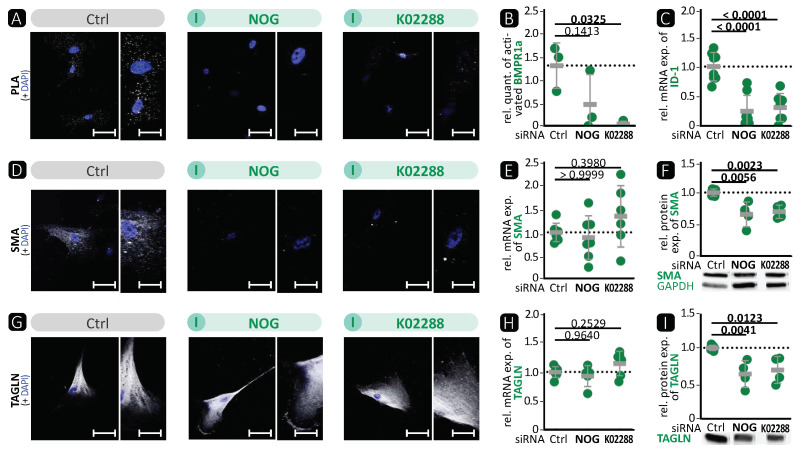
BMP inhibitors noggin or K02288 led to reduced BMP pathway activation and loss of the contractile VSMC phenotype. Human VSMCs were incubated with BMP inhibitors noggin (NOG; 100 ng/mL) or K02288 (1 µM) in 0.4% FBS/EBM (Ctrl). Experiments were performed 72 h post treatment. Data are presented as mean ± SD, and comparisons were calculated by ordinary one-way ANOVA-test with Bonferroni correction for multiple testing; *p*-values as indicated. Rel. = relative; exp. = expression. (**A**,**D**,**G**) Representative photomicrographs of (**A**) in-situ PLA for the detection of BMPR1a phosphorylation after additional stimulation 10 min before analysis and immunostaining against (**D**) SMA and (**G**) TAGLN. DAPI stain visualises nuclei (blue). Left image: scale bar 50 µm; right image: zoomed in to 200%, scale bar 25 µm. (**B**) Relative quantification of PLA-positive dots per DAPI-detected nucleus (*n ≥* 3). (**C**,**E**,**H**) qRT-PCR of (**C**) ID-1 (*n ≥* 5), (**E**) SMA (*n ≥* 5), and (**H**) TAGLN (*n ≥* 4). (**F**,**I**) Western blot analysis of (**F**) SMA (*n ≥* 4) and (**I**) TAGLN (*n ≥* 4) with GAPDH as loading control. Representative blots of GAPDH shown with (**F**).

In order to determine the overall effect of BMP pathway inhibition on VSMCs, we silenced BMP-2 and BMP-4 simultaneously. Additionally, we used the natural compound noggin or the synthetic compound K02288, both known to inhibit BMPR activation [15,38]. The expression after 72 h is shown below; data regarding the effect after 48 h can be found in the Supplementary Materials (Figure S9). We confirmed a robust reduction in BMP-2 and BMP-4 expression (Figure 6A–E). BMPR1a phosphorylation and subsequent ID-1 expression were strongly reduced after siRNA transfection or treatment of VSMC with noggin or K02288 compared to control treatment (Figure 6F–H and Figure 7A–C). For SMAD-6, no consistent significant changes in expression levels were detectable (Figure S9G,T).

In addition, the contractile VSMC marker SMA was significantly reduced by BMP-2/-4 deficiency (Figure 6J,K). Immunocytochemical staining showed that the expression of SMA and TAGLN was reduced (Figure 6I,L). Phalloidin-based staining of the actin cytoskeleton demonstrated lamellipodia with a highly concentrated actin network under the cell borders and additional conglomerates of actin in the cell body instead of complete contractile fibres (Figure 6O). In line with the reduced contractile marker expression, we found that BMP-2/-4 silencing resulted in a strong reduction in cell stiffness and faster relaxation, as assessed by nanoindentation (Figure 6P). Finally, this led to significant functional changes evidenced by a reduced ability to contract collagen and a higher number of migrated VSMCs in BMP-2/-4-deficient VSMCs (Figure 6Q,R). We obtained similar results for the expression of contractile markers using the BMP inhibitors noggin or K02288 for immunocytochemistry and Western blot analysis (Figure 7D–I). 

Taken together, inhibition of the BMP signalling pathway, either by co-inhibition with siRNAs against BMP-2/-4 or with BMP inhibitors such as noggin or K02288, resulted in diminished BMP pathway activity, reduced expression of contractile VSMC markers, and, consequently, reduced contractile VSMC function. These in-vitro results of the human aortic VSMC phenotype after BMP-4 inhibition, showing a synthetic phenotype, are comparable to the results obtained in in-vivo experiments.

## 4. Discussion

Our study indicates that BMP-4 deficiency in VSMCs results in a synthetic phenotype with reduced contractile fibres both in vitro and in vivo. This, in turn, can enhance the development of AngII-induced TAAs. The aortas of BMP-4-deficient mice showed lower expression of contractile markers, while the expression of COL3A1 in IHC and MMP-9 was higher compared to phenotypic wild-type littermates. In-vitro, compensatory effects between BMP-2 and BMP-4 were observed. Although deficiency of BMP-4 resulted in a reduction in contractile markers, the expression of BMP-2 and canonical BMP signalling pathway markers were elevated. Interestingly, when BMP-2/-4 were absent simultaneously or when the BMP pathway inhibitors noggin or K02288 were administered, both the BMP signalling pathway and the contractile VSMC phenotype were suppressed.

BMP-4 specific deletion in VSMCs showed similar characteristics as the AngII-induced TAAs (Figure 2, Figure 3 and Figures S3, and S5). We observed a significant reduction in the expression of SMA, TAGLN, and MYH-11 in the media of VSMCΔBMP-4 mice, which, combined with the unchanged apoptosis rate in the TUNEL assay, does not suggest a depletion of VSMCs at this time point but rather a phenotypic modulation of VSMCs, which we also observed in the in-vitro assays. As previously described by others, this switch from a contractile to a synthetic phenotype in VSMCs usually occurs early in experimental models, before visible damage in aneurysm formation appears [14]. It is assumed that the transition of VSMCs to a dedifferentiated phenotype leads to higher MMP-9 expression and activation, which can cause elastin destruction [14]. Additionally, MMPs regulate the proliferation and migration of VSMCs by various mechanisms, including the degradation of the ECM and the activation of growth factors (such as TGF-β) through proteolytic cleavage, which promotes migration [39]. We also observed a migratory phenotype in BMP-4-deficient VSMCs, and migration was increased in vitro when BMP-4 was deficient together with the compensatory BMP-2. In conclusion, we found that BMP-4 plays an important role in maintaining balance in the aortic wall, as its absence paves the way for TAA development.

There is strong evidence that TGF-β plays a central role in the development of TAAs, including AngII-induced TAAs [40,41]. However, the impacts of TGF-β on TAAs are contradictory, as both higher and lower activity of TGF-β in TAAs have been observed [42]. We hypothesised that BMP-4 plays a crucial role in addition to TGF-β during the development of TAAs. There is a clear distinction between TGF-β and BMP-2/-4 signalling, as the target protein of the canonical BMP pathway, ID-1 [43], cannot be activated by TGF-β [44]. However, Jones et al. [45] showed in a surgical elastase TAA mouse model that the expression of members of the BMP pathway is indeed changed. Interestingly, we observed that loss of BMP-4 in VSMCs in the AngII-induced TAA mouse model resulted in stronger aortic wall destruction with enhanced MMP-9 expression and activity as well as more apoptosis compared to AngII treatment itself. Taken together, this is the first report demonstrating that the loss of BMP-4 in VSMCs promotes TAA development. 

We and others [19] have shown that BMP-4 promotes the contractile VSMC phenotype (Figure 1). Therefore, we hypothesised that, vice versa, BMP-4 deficiency would diminish the contractile VSMC phenotype in vitro. Indeed, immunofluorescence staining of SMA or TAGLN in VSMCs revealed a migratory phenotype (Figure 4 and Figure S7). Overall, no quantitative reduction was detected in the protein levels of contractile markers. However, the cytoskeleton in the cells appeared altered, indicating a modification that occurs during the transformation of the contractile VSMCs to a migrating cell [46]. Moreover, the significant reduction in contractility in the collagen gel contraction assay confirms that a loss of the contractile phenotype is occurring. Corriere et al. [47] showed that BMP-4 reduces the migration of VSMCs, which is also consistent with a contractile phenotype enhanced by BMP-4. However, despite BMP-4 deficiency and a demonstrated shift to a dedifferentiated phenotype, we did not detect enhanced migration in BMP-4-deficient VSMCs. Collectively, we observed that silencing BMP-4 in human aortic VSMCs reduces the contractile phenotype, as evidenced by reduced contraction and rearrangement of the contractile apparatus into a pre-migratory phenotype.

Although BMP-2 and BMP-4 belong to the same subset of the TGF-β family, and their amino acid sequence is more than 86% similar, they are transcribed from two entirely different genes with markedly different promoter regions and regulatory elements [37,48]. It has already been shown that BMP-2 reduces the expression of contractile markers in VSMCs, while BMP-4 elevates them. This effect is mediated through a consensus TGF-β-controlling element, which is present in the regulatory regions of many contractile marker genes [49,50]. In our experiments, the expression of ID-1 as target of the canonical signalling pathway correlated well with the phosphorylation status of the upstream receptor BMPR1a. This suggests that the signalling pathway of BMP-2 and BMP-4 via the BMPR1a receptor primarily activates the canonical signalling pathway. Surprisingly, we detected that silencing of BMP-4 in VSMCs elevates the BMP pathway activity upstream with enhanced phosphorylation of BMPR1a (Figure 4E). We hypothesise that the stronger activation of the pathway is related to the compensatory increase in BMP-2 mRNA expression (Figure 4). This hypothesis is supported by the fact that BMP-2 deficiency also reduced BMP pathway activation (Figure 5), suggesting that canonical signalling is dominated by BMP-2 over BMP-4. On the other hand, we were able to show that BMP-2 deficiency led to a pronounced contractile phenotype, as evidenced by a significantly higher protein expression of SMA and TAGLN and a denser cytoskeletal network. Therefore, we assume that the influence of BMP-4 on the contractile phenotype is stronger than that of BMP-2. Moreover, it appears that the SMAD-dependent canonical signalling pathway is unable to control the expression of contractile markers. This implies the involvement of other non-canonical signalling pathways. One such pathway is the ERK pathway, which has been shown to be stimulated by BMP-4 and to promote the contractile phenotype [51,52]. 

In-vitro, BMP-4 deficiency showed a phenotype consistent with the in-vivo experiments. In both models, the deficiency resulted in a loss of the contractile phenotype, which could promote the development of aortic aneurysms. Figure 4 shows an increase in the activation of the BMP pathway, whereas the opposite was observed in VSMCΔBMP-4 mice. This illustrates the complexity of in-vivo regulation by the interaction of different cell types and suggests the involvement of additional pathways that may prevent or suppress the compensatory increase in BMP-2. Further investigations are required to determine the role of BMP-2 and other possible signalling pathways. Interestingly, in BMP-2/-4 double deficiency, both BMP-2-induced canonical BMP pathway activation and BMP-4-promoted contractile VSMC phenotype were reduced (Figure 6). This indicates the initiation of a fully dedifferentiated VSMC phenotype. This was further supported by the phalloidin staining, which revealed lamellipodia with a leading edge representing the epicentre of cytoskeleton activity necessary for cell migration [46]. The notion of VSMC dedifferentiation was further supported by the observation that cell stiffness and relaxation were markedly reduced in the nanoindentation experiments. Additionally, the complete abolition of VSMC contractility and significantly higher migration rate are typical characteristics of a switch to the synthetic VSMC phenotype. Similar results were obtained when VSMCs were treated with the BMP inhibitors noggin or K02288. Noggin blocks both BMP epitopes, preventing any interaction of BMP family members with cell surface receptors [53]. K02288, on the other hand, only inhibits the activin receptor-like kinases (ALK) ALK-1, ALK-2, BMPR1a (ALK-3), and BMPR1b (ALK-6) [38]. Since both inhibitors induced the loss of the contractile VSMC phenotype comparable to BMP-2/-4 double deficiency, our findings suggest that the effects of BMP-2 and BMP-4 on the contractile phenotype of VSMCs are mediated through one of the ALK family receptors. We found that the phosphorylation of BMPR1a did not correlate with the expression of contractile proteins and that ALK-1 is mainly expressed on endothelial cells [21]. It is tempting to speculate that the signal may be transmitted via ALK-2 or BMPR1b [21]. Taken together, inhibition of the BMP pathway in human aortic VSMCs, either by silencing BMP-2/-4 or by inhibition through noggin or K02288, clearly promotes the phenotypic switch from the contractile to the synthetic VSMC phenotype. 

The study has several limitations. For protein and mRNA analyses from aortic tissue, complete purity regarding intimal and adventitial tissue could not be technically controlled, despite the highest accuracy in isolating VSMCs. Additionally, cell culture is limited by the fact that VSMCs tend to exhibit a dedifferentiated phenotype when cultured and often fail to adequately recapitulate regulatory pathways that are critical in vivo. Multiple differentiation markers are necessary, and therefore included here, to characterise the specific phenotype of cultured VSMCs due to the inability of a single marker to distinguish VSMCs in each passage or from other cell types. Mice were only analysed at a single time point after 4 weeks of AngII infusion. Therefore, no statement about temporal effects of BMP4 silencing can be drawn, necessitating further studies to address the temporal dynamics/compensation following BMP silencing. In addition, the transfer of our in-vitro data regarding the effects of BMP-2 to a genetic model including single- and double-deficient BMP-2/-4-deficient mice is currently lacking. Nevertheless, the data provided so far hopefully open a broad field of opportunities for more in-depth investigations.

In conclusion, the data presented in this study demonstrate the significance of BMP-4 in maintaining the contractile phenotype of VSMCs. The loss of BMP-4 in aortic VSMCs contributes to the development of TAAs. In addition, we were able to show that despite their homology, BMP-2 and BMP-4 have different effects and are expressed in a compensatory manner. Furthermore, they function differently in VSMCs. BMP-2 appears to predominantly determine the canonical BMP signalling pathway. In contrast, BMP-4 appears to have a greater impact on the contractile phenotype than BMP-2. Together, this indicates that BMP-4 most likely signals via alternative non-SMAD-dependent pathways such as the MAPK pathway, as it is well known that ERK signalling also increases the expression of contractile markers [51,52]. 

To the best of our knowledge, our study is the first to report the loss of BMP-4 function in vitro and in vivo in aortic VSMCs, which may contribute to the development of TAAs. Further investigation is necessary to better understand the complex interplay during the genesis of TAAs and the mechanisms by which BMP-4 influences contractile markers independently of the canonical pathway. Furthermore, a comprehensive examination of the BMP pathway and its various subgroup BMP ligands, including BMP-6/-7 and BMP-9/-10, during TAA development is currently lacking. As a result, our research provides the initial foundation for future investigations into the BMP signalling pathway in aortic diseases. Overall, our study highlights the necessity for a more thorough analysis of the BMP signalling pathway during TAA development to identify potential therapeutic targets.

## Data Availability

The data presented in this study are available on request from the corresponding author.

## Supplementary Materials

The following supporting information can be downloaded at: https://www.mdpi.com/article/10.3390/cells13090735/s1, Figure S1: Supplementary data to Figure 1; Figure S2: Analysis of the impact of Cre activity in VSMCs in the MYH-11CreERT2 model; Figure S3: Supplementary data to Figure 2; Figure S4: Isolated VSMCs of mice show a lower expression of contractile markers; Figure S5: Supplementary data to Figure 3; Figure S6: Echocardiographic studies analysing the effect of BMP-4 deficiency on cardiac function with and without Angiotensin II influence; Figure S7: Supplementary data to Figure 4; Figure S8: Supplementary data to Figure 5; Figure S9: Supplementary data to Figure 6 and Figure 7; Figure S10: Negative controls of immunocytological and immunohistological stainings; Figure S11: Full unedited Western blots; Major Resources Table; ARRIVE Guidelines.
