# Supplementary material for: Divergent and Compensatory Effects of BMP2 and BMP4 on the VSMC Phenotype and BMP4’s Role in Thoracic Aortic Aneurysm Development
Source: Cells. 2024 Apr 24;13(9):735. doi: 10.3390/cells13090735 (PMC11083443; doi:10.3390/cells13090735)
Supplement: Supplementary file 1 [file cells-13-00735-s001.zip › cells-2975109-supplementary.pdf]

SUPPLEMENTAL FIGURES

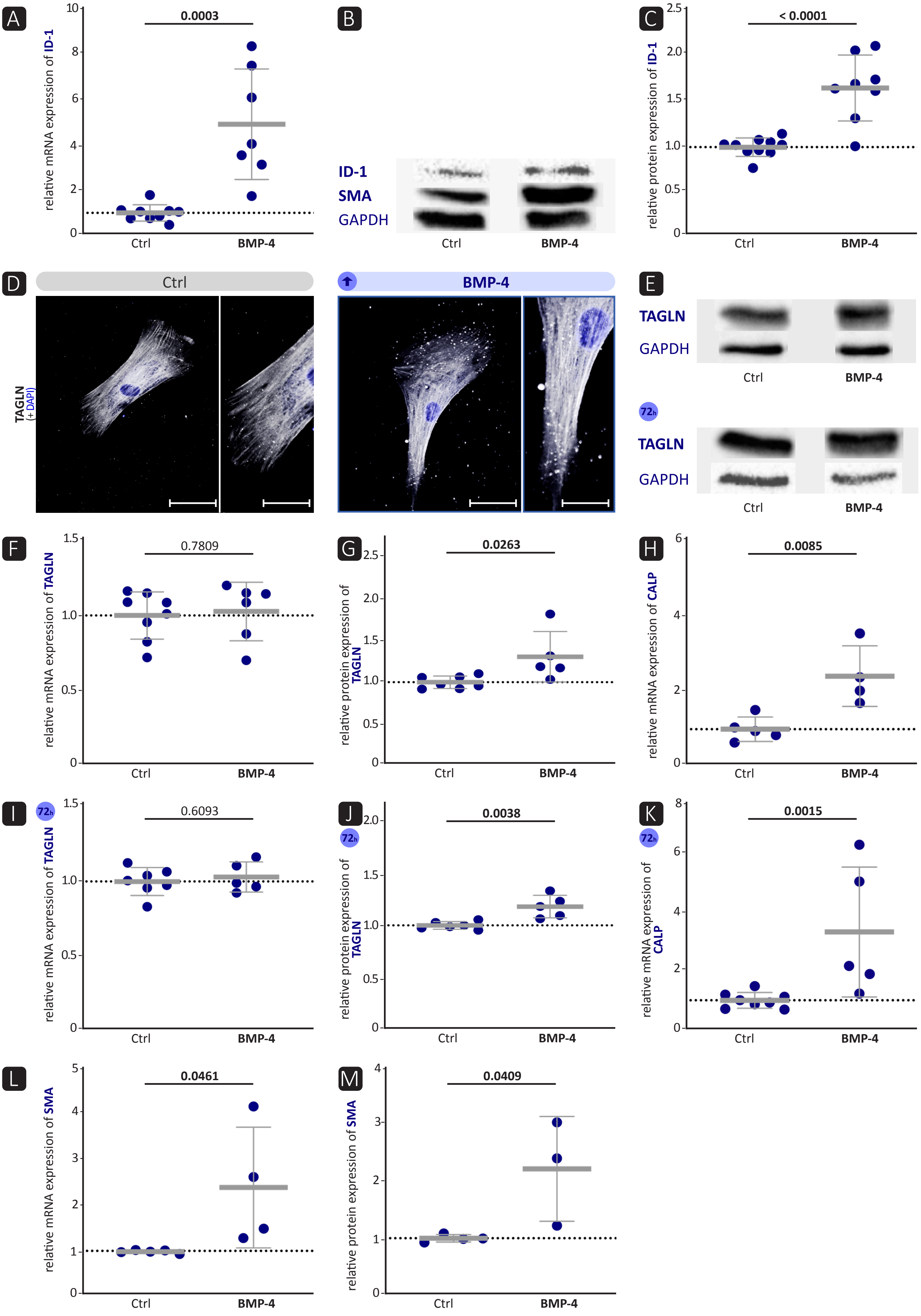
A
B
C
D
E
F
G
H
I
J
K
L
M
0.0003
< 0.0001
relative mRNA expression of ID-1
relative protein expression of ID-1
ID-1
SMA
GAPDH
Ctrl
BMP-4
TAGLN
(+DAPI)
72h
0.7809
0.0263
0.0085
0.6093
0.0038
0.0015
0.0461
0.0409
relative mRNA expression of TAGLN
relative protein expression of TAGLN
relative mRNA expression of CALP
relative mRNA expression of SMA
relative protein expression of SMA

Figure S1: Supplementary data to figure 1. Human VSMC were either incubated in 0.4% FBS/EBM (Ctrl) or stimulated with BMP-4 (40 ng/mL). Experiments were performed after 48 hours of treatment unless otherwise indicated. Data are presented as mean±SD and comparisons were calculated by an unpaired Student's t-test; p-values as indicated. (**A+F+H+I+K+L**) qRT-PCR of (A) ID-1 (n≥7), (F) TAGLN (n≥6), (H) CALP (n≥4), (I) TAGLN (n≥5), (K) CALP (n≥5) and (L) SMA (n≥4). (**B+C+E+G+J+M**) Western blot analysis of (C) ID-1 (n≥8), (G) TAGLN (n≥5), (J) TAGLN (n≥5) and (M) SMA (n≥3) with GAPDH as loading control. Representative blots in B and E. (**D**) Representative photomicrographs of immunofluorescence staining for TAGLN after 72 hours. Nuclei were stained with DAPI (blue). Left image: scale bar 50 μm; right image: zoom in to 200%, scale bar 25 μm.

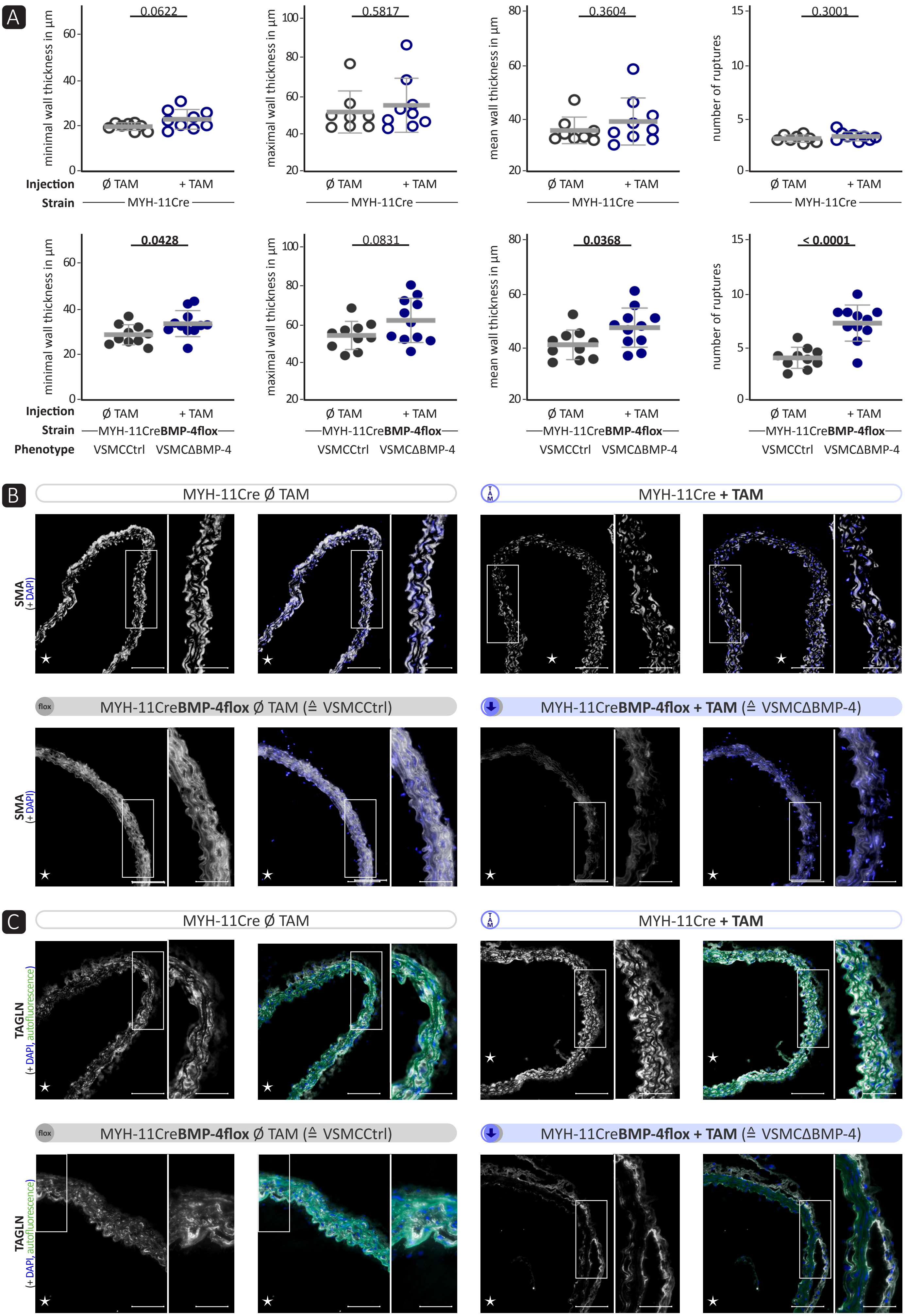
A
minimal wall thickness in µm
maximal wall thickness in µm
mean wall thickness in µm
number of ruptures
0.0622
0.5817
0.3604
0.3001
Injection
Strain
Ø TAM
+ TAM
MYH-11Cre
0.0428
0.0831
0.0368
< 0.0001
Phenotype
MYH-11CreBMP-4flox
VSMCCtrl
VSMCΔBMP-4
B
MYH-11Cre Ø TAM
MYH-11Cre + TAM
SMA (+ DAPI)
MYH-11CreBMP-4flox Ø TAM (≙ VSMCCtrl)
MYH-11CreBMP-4flox + TAM (≙ VSMCΔBMP-4)
C
TAGLN (+ DAPI, autofluorescence)

Figure S2: Analysis of the impact of Cre-activity in VSMC in the MYH-11Cre^{ERT} model. Six to eight weeks old male MYH-11Cre or MYH-11CreBMP-4flox mice, were injected intraperitoneally with tamoxifen (+ TAM) over 5 days. At 16-18 weeks after the last injection, the aorta was isolated. Mice of the same genotype without tamoxifen injection (∅ TAM) served as controls. Data are presented as mean±SD and comparisons were calculated by an unpaired Student's t-test; p-values as indicated. (**A**) Quantification of the Elastica van Gieson staining of thoracic aortic tissue sections. Analysis of minimal, maximal and mean wall thickness and number of ruptures (n≥7). (**B+C**) Representative photomicrographs of cross-sections of thoracic aortic tissue with immunostaining against (B) SMA, (C) TAGLN. Left image: scale bar 100 µm; right image: zoomed to 200% of the highlighted area, scale bar 50 µm. Vascular lumen is marked with an asterisk. DAPI stain visualizes nuclei (blue). Elastic fibres in green autofluorescence.

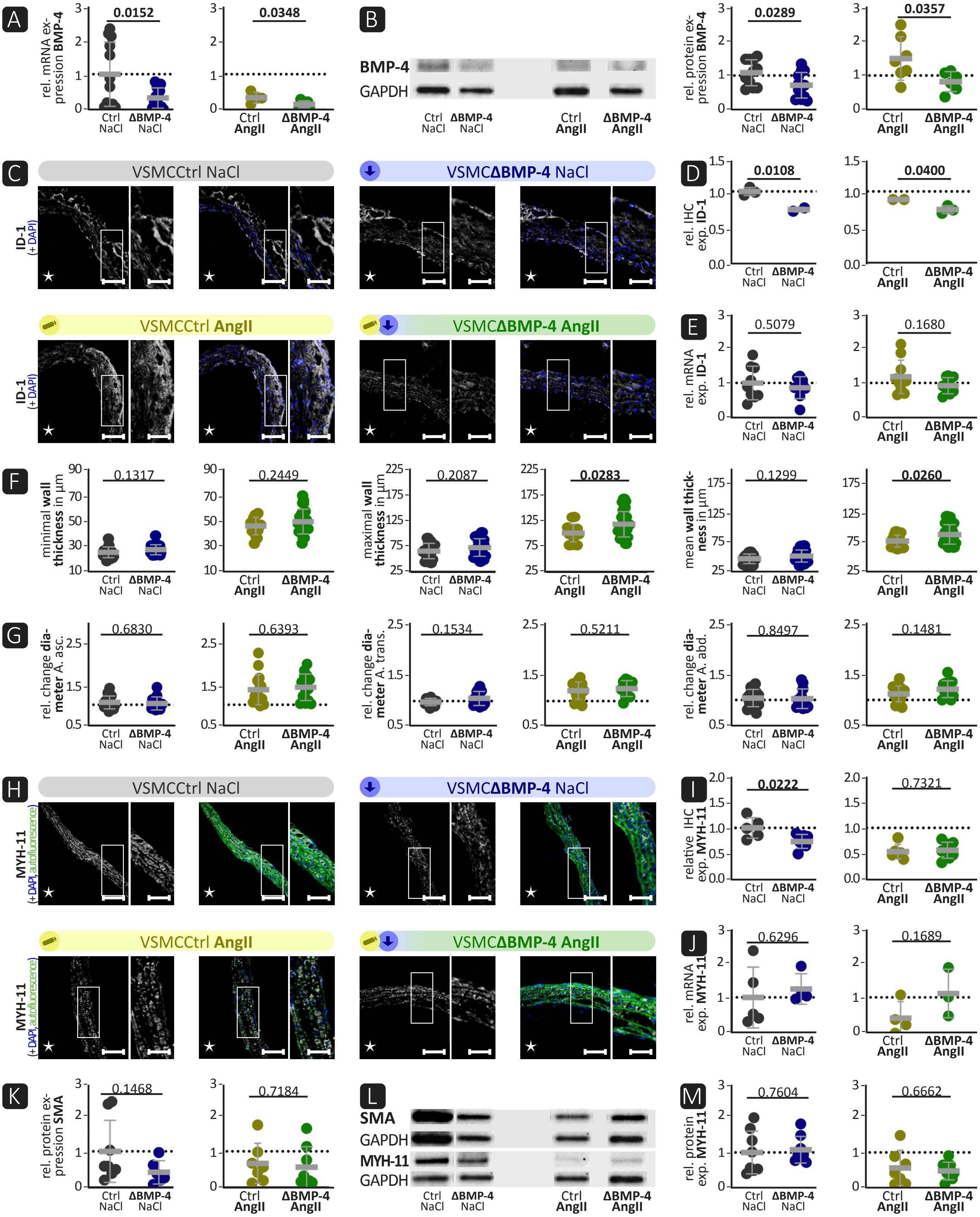
A
B
C
D
E
F
G
H
I
J
K
L
M
BMP-4
GAPDH
SMA
MYH-11
VSMCCtrl NaCl
VSMCΔBMP-4 NaCl
VSMCCtrl AngII
VSMCΔBMP-4 AngII
ID-1 (+DAPI)
MYH-11 (+DAPI, autofluorescence)
rel. mRNA expression BMP-4
rel. protein expression BMP-4
rel. IHC exp. ID-1
rel. mRNA exp. ID-1
minimal wall thickness in µm
maximal wall thickness in µm
mean wall thickness in µm
rel. change diameter A. asc.
rel. change diameter A. trans.
rel. change diameter A. abd.
relative IHC exp. MYH-11
rel. mRNA exp. MYH-11
rel. protein expression SMA
rel. protein exp. MYH-11
Ctrl NaCl
ΔBMP-4 NaCl
Ctrl AngII
ΔBMP-4 AngII
0.0152
0.0348
0.0289
0.0357
0.0108
0.0400
0.5079
0.1680
0.1317
0.2449
0.2087
0.0283
0.1299
0.0260
0.6830
0.6393
0.1534
0.5211
0.8497
0.1481
0.0222
0.7321
0.6296
0.1689
0.1468
0.7184
0.7604
0.6662

Figure S3: Supplementary data to figure 2 A subcutaneous pump with AngII (flow rate = 1 µg/kg/min) or 0.9% NaCl was implanted in phenotypic wild-type littermates VSMCCtrl (Ctrl) or VSMCΔBMP-4 mice (ΔBMP-4) 28 days before the thoracic aorta was isolated. Data are presented as mean±SD and comparisons were calculated by an unpaired Student's t-test; p-values as indicated. Rel.=relative; exp.=expression. (**A+E+J**) qRT-PCR of (A) BMP-4 (n≥4), (E) ID-1 (n≥4), (J) MYH-11 (n≥4). (**B+K+L+M**) Western blots analyses of (B) BMP-4 (n≥4), (K) SMA (n≥5), (M) MYH-11 (n≥5) with GAPDH as loading control. Representative blots in B and L. (**C+H**) Representative photomicrographs of immunostaining against (C) ID-1, (H) MYH-11. Vascular lumen is marked with an asterisk. Left image: scale bar 100 µm; right image: zoomed to 200% of the highlighted area, scale bar 50 µm. DAPI stain visualized nuclei (blue) in immunohistological stainings. Autofluorescence of elastic fibers is in green when indicated in the figure. (**D+I**) Quantification of mean density of IHC of (D) ID-1 (n≥3), (I) MYH-11 (n≥3). (**F**) Change over 28 days in minimal, maximal and mean wall thickness (n≥10). (**G**) Relative change over 28 days in aortic diameter of the aorta ascendens (A. asc.), aorta transversalis (A. trans.) and aorta abdominalis (A. abd.) (n≥17).

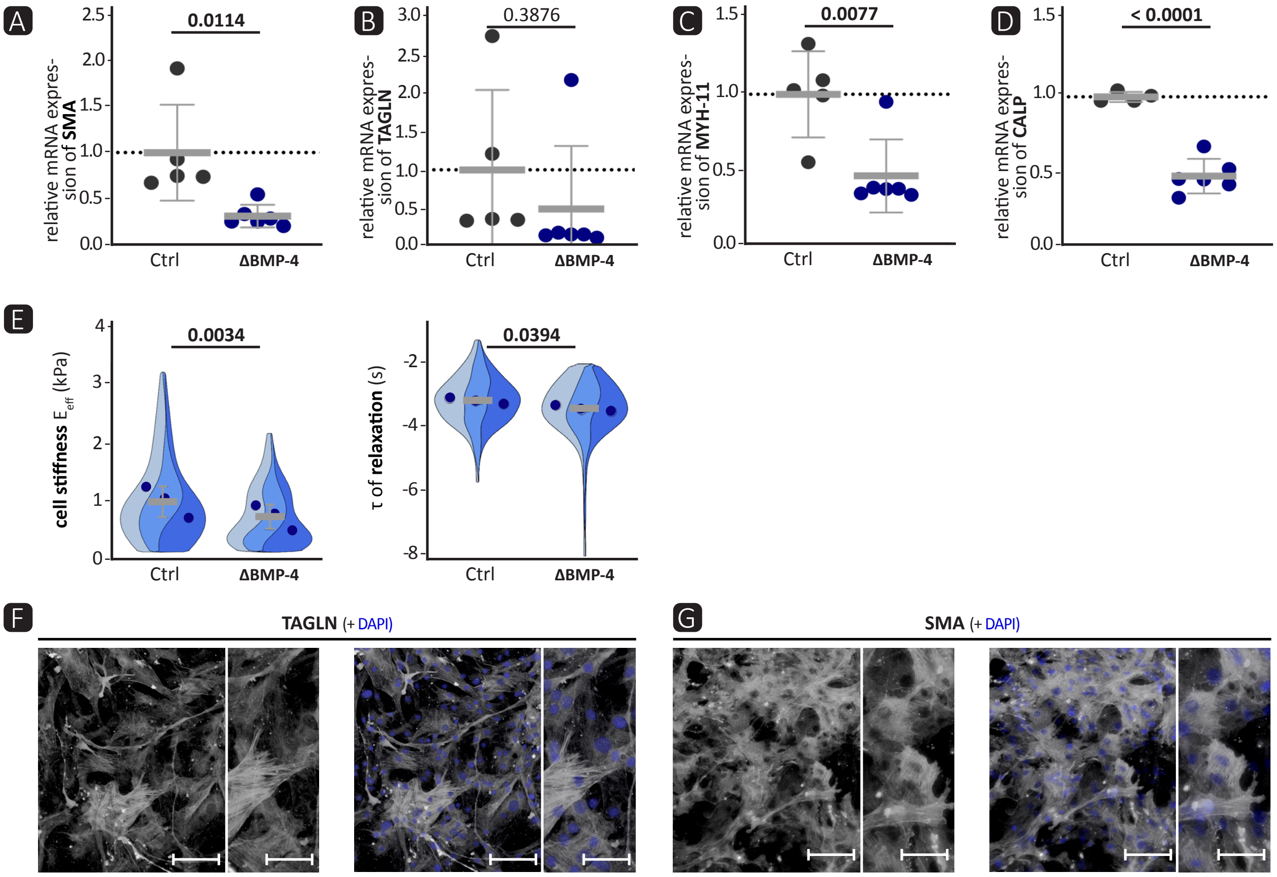
A
relative mRNA expres-sion of SMA
0.0114
Ctrl
ΔBMP-4
B
relative mRNA expres-sion of TAGLN
0.3876
C
relative mRNA expres-sion of MYH-11
0.0077
D
relative mRNA expres-sion of CALP
< 0.0001
E
cell stiffness E_eff (kPa)
0.0034
τ of relaxation (s)
0.0394
F
TAGLN (+ DAPI)
G
SMA (+ DAPI)

Figure S4: Isolated aortic mouse VSMC with BMP-4 deficiency show a lower expression of contractile markers. Four to six weeks old male VSMCCtrl (Ctrl) or VSMCΔBMP-4 mice (ΔBMP-4) were used. At 10 days after the last injection, the aorta was isolated and VSMC were extracted by enzymatic digestion. Data are presented as mean±SD and comparisons were calculated by an unpaired Student's t-test; p-values as indicated. (**A+B+C+D**) qRT-PCR of (A) SMA (n≥5), (B) TAGLN (n≥5), (C) MYH-11 (n≥5) and (D) CALP (n≥4) of isolated murine VSMC. (**E**) Nanoindentation: Effective Young's modulus E_{eff} and time constant τ of relaxation of isolated VSMCΔBMP-4 vs Ctrl (3 experiments, each with n≥13). (**F+G**) Representative photomicrographs of immunostaining against (F) SMA and (G) TAGLN to verify culture purity of isolated murine VSMC. Left image: scale bar 100 μm; right image: zoom in to 200%, scale bar 50 μm. Nuclei were stained with DAPI (blue).

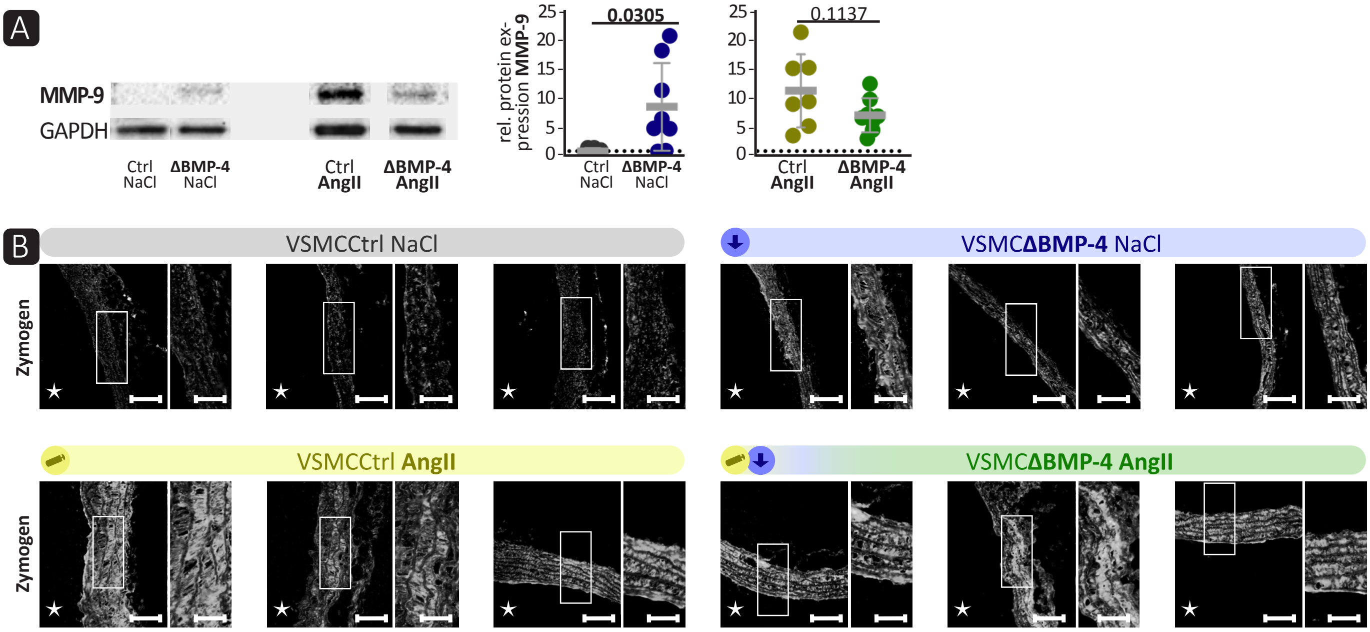
A
MMP-9
GAPDH
Ctrl NaCl
ΔBMP-4 NaCl
Ctrl AngII
ΔBMP-4 AngII
rel. protein expression MMP-9
0.0305
0.1137
B
VSMCCtrl NaCl
VSMCΔBMP-4 NaCl
VSMCCtrl AngII
VSMCΔBMP-4 AngII
Zymogen

Figure S5: Supplementary data to figure 3 A subcutaneous pump with AngII (flow rate = 1 μg/kg/min) or 0.9% NaCl was implanted in phenotypic wild-type littermates VSMCCtrl (Ctrl) or VSMCΔBMP-4 mice (ΔBMP-4) 28 days before the thoracic aorta was isolated. (**A**) Western blot analysis of MMP-9 ($n \geq 7$) with GAPDH as loading control. Data are presented as mean±SD and comparisons were calculated by an unpaired Student's t-test; p-values as indicated. (**B**) Representative photomicrographs of zymogen assay from three different mice. Vascular lumen is marked with an asterisk. Left image: scale bar 100 μm; right image: zoomed to 200% of the highlighted area, scale bar 50 μm. DAPI stain visualized nuclei (blue) in immunohistological stainings.

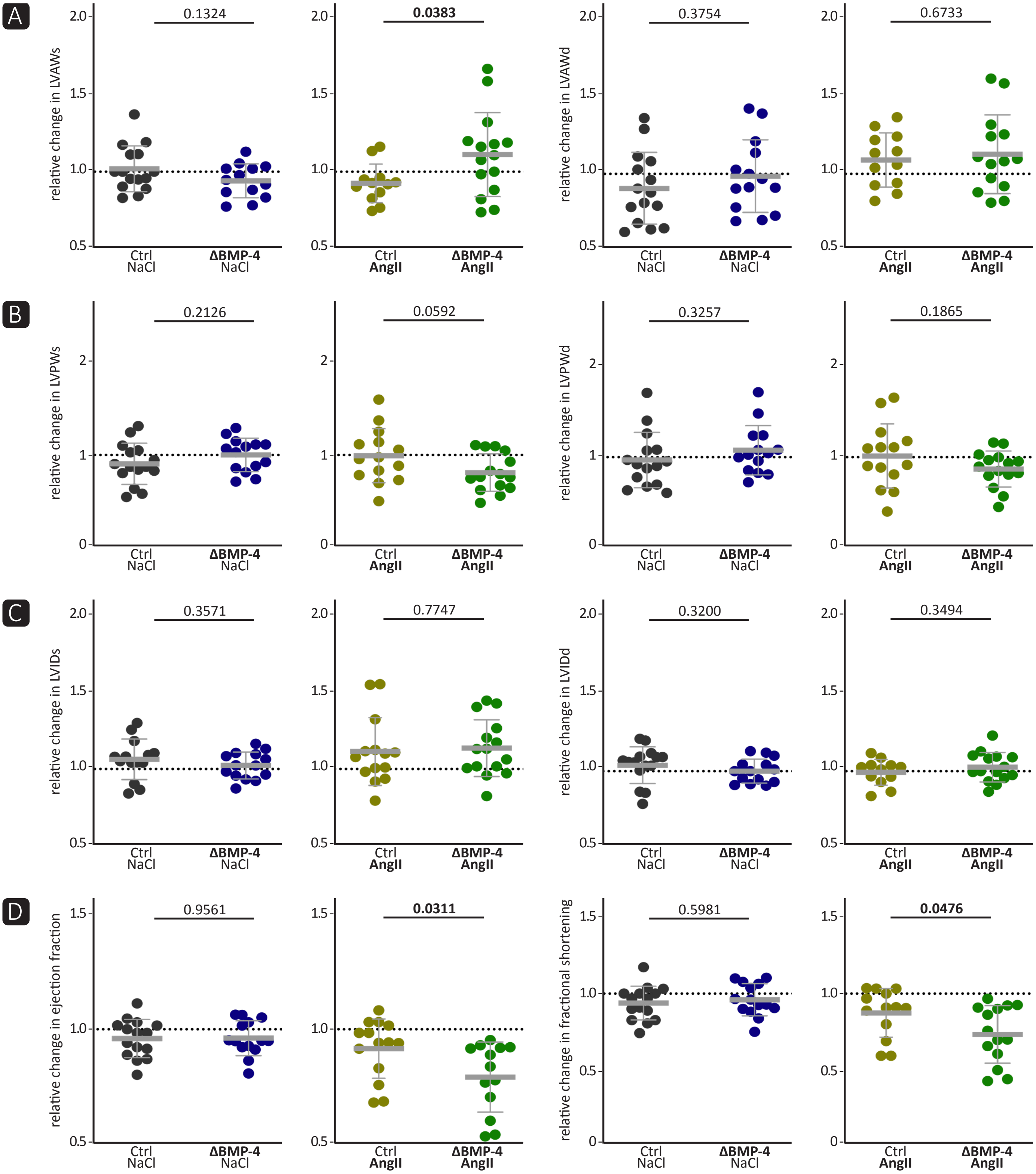
A
B
C
D
relative change in LVAWs
relative change in LVAWd
relative change in LVPWs
relative change in LVPWd
relative change in LVIDs
relative change in LVIDd
relative change in ejection fraction
relative change in fractional shortening
0.1324
0.0383
0.3754
0.6733
0.2126
0.0592
0.3257
0.1865
0.3571
0.7747
0.3200
0.3494
0.9561
0.0311
0.5981
0.0476
Ctrl NaCl
ΔBMP-4 NaCl
Ctrl AngII
ΔBMP-4 AngII

Figure S6: Echocardiographic studies analysing the effect of BMP-4 deficiency on cardiac function with and without Angiotensin II influence. A subcutaneous pump with AngII (flow rate = 1 µg/kg/min) was implanted in phenotypic wild-type littermates VSMCCtrl (Ctrl) or VSMCΔBMP-4 mice (ΔBMP-4) and echocardiography was performed. After 28 days a second echo was performed, and the heart was isolated. Data are presented as mean±SD and comparisons were calculated by an unpaired Student's t-test; p-values as indicated. (**A**) Relative change over 28 days of left ventricular anterior wall thickness end systole (LVAWs) and end diastole (LVAWd) (n≥15). (**B**) Relative change over 28 days of left ventricular posterior wall thickness end systole (LVPWs) and end diastole (LVPWd) (n≥15). (**C**) Relative change over 28 days of left ventricular internal diameter end systole (LVIDs) and end diastole (LVIDd) (n≥15). (**D**) The ejection fraction (EF) and fractional shortening (FS) were calculated from LVIDs and LVIDd: EF (%) = $((LVIDd^3 - LVIDs^3) / LVIDd^3) * 100$; FS (%) = $((LVIDd - LVIDs) / LVIDd) * 100$. Relative change over 28 days is shown (n≥16).

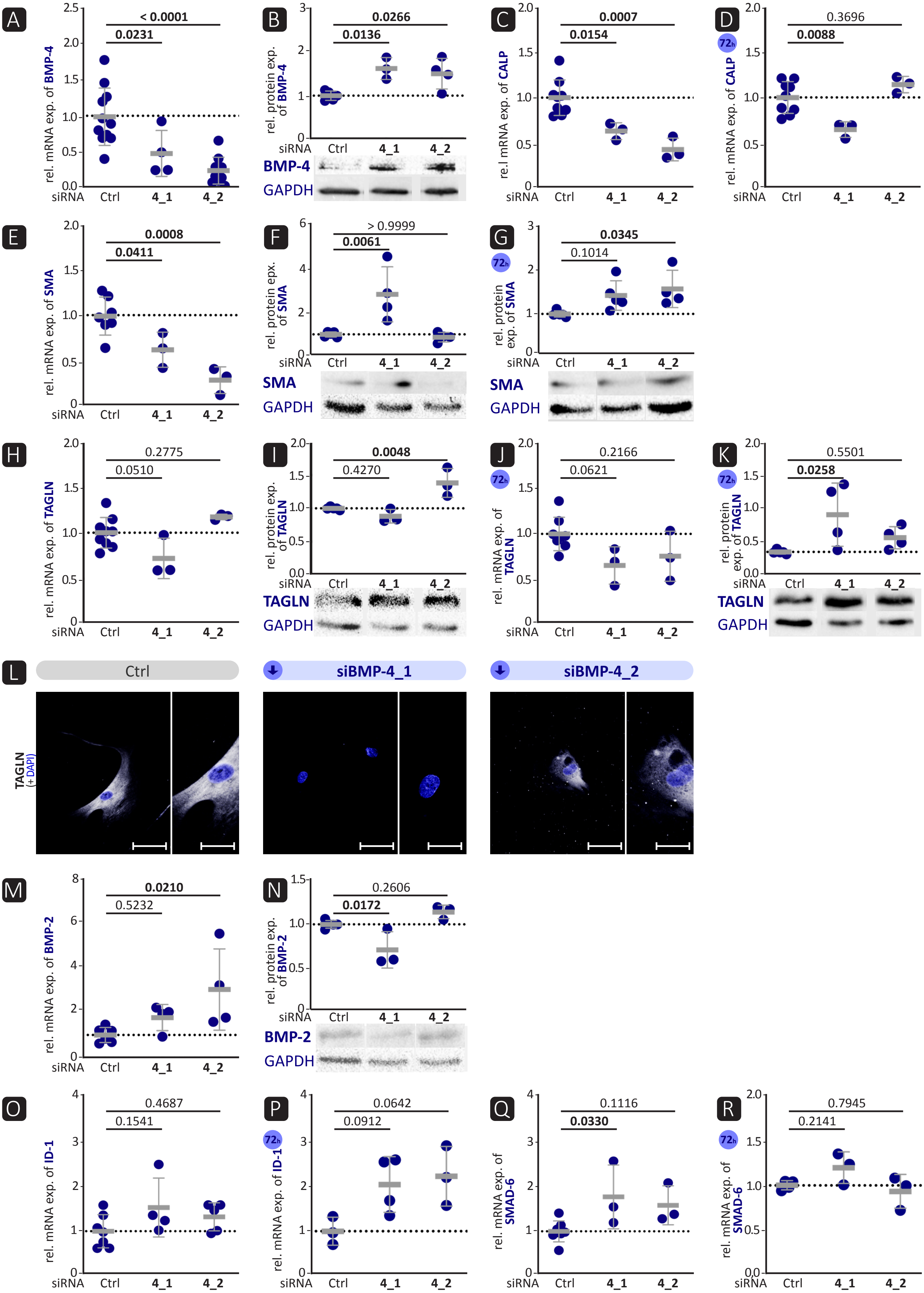
A
rel. mRNA exp. of BMP-4
< 0.0001
0.0231
siRNA Ctrl 4_1 4_2
B
rel. protein exp. of BMP-4
0.0266
0.0136
BMP-4
GAPDH
C
rel. mRNA exp. of CALP
0.0007
0.0154
D
72h
rel. mRNA exp. of CALP
0.3696
0.0088
E
rel. mRNA exp. of SMA
0.0008
0.0411
F
rel. protein epx. of SMA
> 0.9999
0.0061
SMA
GAPDH
G
72h
rel. protein exp. of SMA
0.0345
0.1014
H
rel. mRNA exp. of TAGLN
0.2775
0.0510
I
rel. protein exp. of TAGLN
0.0048
0.4270
TAGLN
GAPDH
J
72h
rel. mRNA exp. of TAGLN
0.2166
0.0621
K
72h
rel. protein exp. of TAGLN
0.5501
0.0258
L
Ctrl
siBMP-4_1
siBMP-4_2
TAGLN (+ DAPI)
M
rel. mRNA exp. of BMP-2
0.0210
0.5232
N
rel. protein exp. of BMP-2
0.2606
0.0172
BMP-2
GAPDH
O
rel. mRNA exp. of ID-1
0.4687
0.1541
P
72h
rel. mRNA exp. of ID-1
0.0642
0.0912
Q
rel. mRNA exp. of SMAD-6
0.1116
0.0330
R
72h
rel. mRNA exp. of SMAD-6
0.7945
0.2141

Figure S7: Supplementary data to figure 4. BMP-4 deficiency was induced in VSMC with either of two specific siRNA targeted against BMP-4 (4_1, 4_2). Scrambled siRNA was used as a control (Ctrl). Experiments were performed 48 hours post-siRNA transfection unless otherwise indicated. Data are presented as mean±SD and comparisons were calculated by an ordinary one-way ANOVA-test with Bonferroni correction for multiple testing; p-values as indicated. Rel.=relative; exp.=expression. (**A+C+D+E+H+J+M+O+P+Q+R**) qRT-PCR of (A) BMP-4 (n≥4), (C+D) CALP (n≥3), (E) SMA (n≥3), (H+J) TAGLN (n≥3), (M) BMP-2 (n≥4), (O+P) ID-1 (n≥4), (Q+R) SMAD-6 (n≥3). (**B+F+G+I+K+N**) Western blot analysis of (B) BMP-4 (n≥3), (F+G) SMA (n≥4), (I+K) TAGLN (n≥3), (N) BMP-2 (n≥3) with GAPDH as loading control. (**L**) Representative photomicrographs of immunostaining against TAGLN 72 hours *post* transfection. Left image: scale bar 50 µm; right image: zoom in to 200%, scale bar 25 µm.

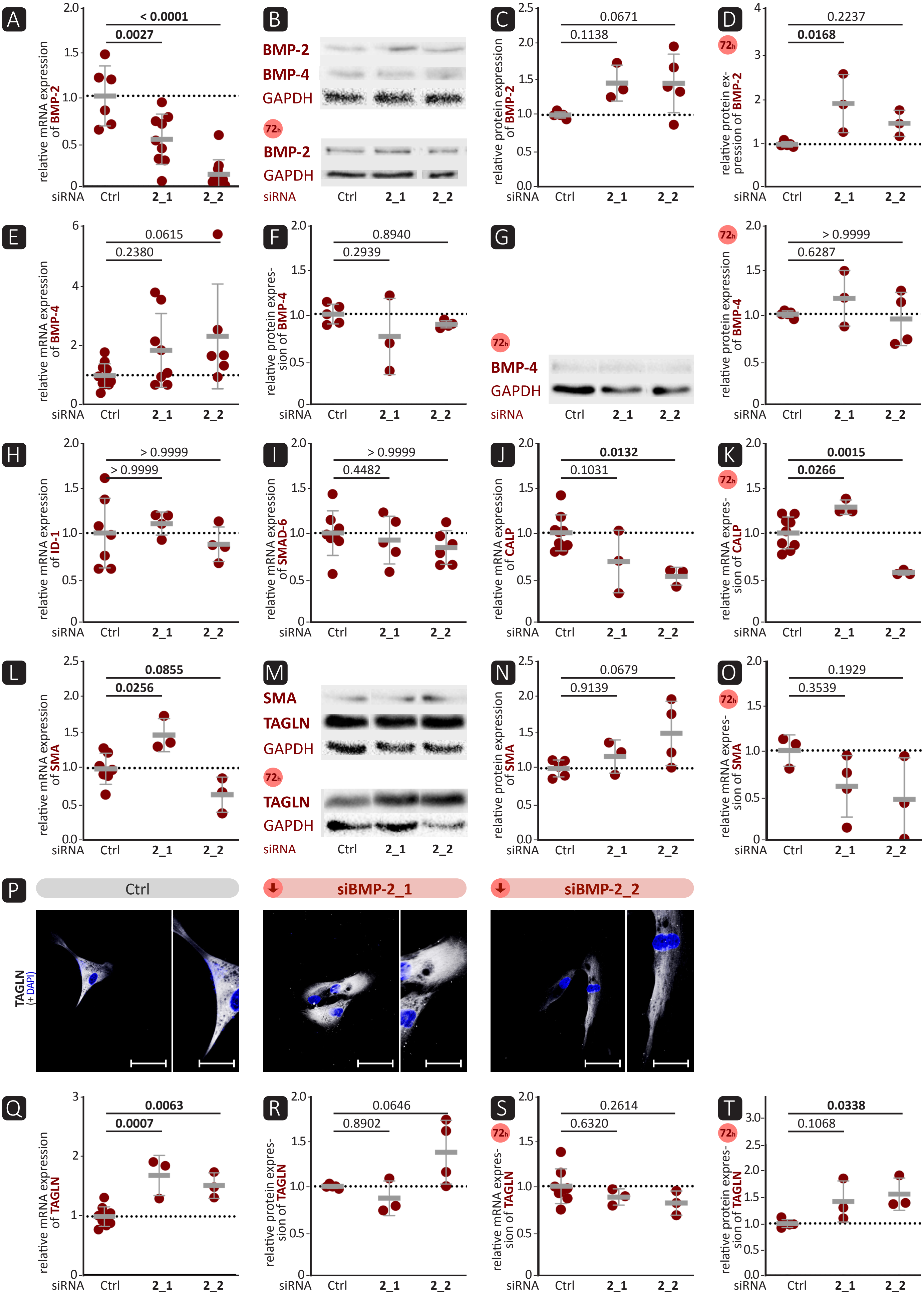
A
B
C
D
E
F
G
H
I
J
K
L
M
N
O
P
Q
R
S
T
< 0.0001
0.0027
relative mRNA expression of BMP-2
BMP-2
BMP-4
GAPDH
72h
siRNA
Ctrl
2_1
2_2
0.0671
0.1138
relative protein expression of BMP-2
0.2237
0.0168
relative protein ex- pression of BMP-2
0.0615
0.2380
relative mRNA expression of BMP-4
0.8940
0.2939
relative protein expres- sion of BMP-4
> 0.9999
0.6287
relative protein expression of BMP-4
0.4482
relative mRNA expression of ID-1
relative mRNA expression of SMAD-6
0.0132
0.1031
relative mRNA expression of CALP
0.0015
0.0266
relative mRNA expres- sion of CALP
0.0855
0.0256
relative mRNA expression of SMA
SMA
TAGLN
0.0679
0.9139
relative protein expression of SMA
0.1929
0.3539
relative mRNA expres- sion of SMA
siBMP-2_1
siBMP-2_2
TAGLN (+ DAPI)
0.0063
0.0007
relative mRNA expression of TAGLN
0.0646
0.8902
relative protein expres- sion of TAGLN
0.2614
0.6320
relative mRNA expres- sion of TAGLN
0.0338
0.1068
relative protein expres- sion of TAGLN

Figure S8: Supplementary data to figure 5. BMP-2 deficiency was induced in human VSMC with either of two specific siRNA targeted BMP-2 (2_1, 2_2). Scrambled siRNA was used as control (Ctrl). Experiments were performed 48 hours *post* siRNA transfection, unless otherwise stated. Data are presented as mean±SD and comparisons were calculated by an ordinary one-way ANOVA-test with Bonferroni correction for multiple testing; p-values as indicated. (**A+E+H+I+J+K+L+O+Q+S**) qRT-PCR of (A) BMP-2 (n≥6), (E) BMP-4 (n≥6), (H) ID-1 (n≥4), (I) SMAD-6 (n≥5), (J+K) CALP (n≥3), (L+O) SMA (n≥3), (Q+S) TAGLN (n≥3). (**B+C+D+F+G+M+N+R+T**) Western blot analysis of (C+D) BMP-2 (n≥3), (F+G) BMP-4 (n≥3), (N) SMA (n≥3), (R+T) TAGLN (n≥3) with GAPDH as loading control. Representative blots in B, G, M. (**P**) Representative photomicrographs of immunostaining against TAGLN 72 hours *post* transfection. Left image: scale bar 50 µm; right image: zoom in to 200%, scale bar 25 µm.

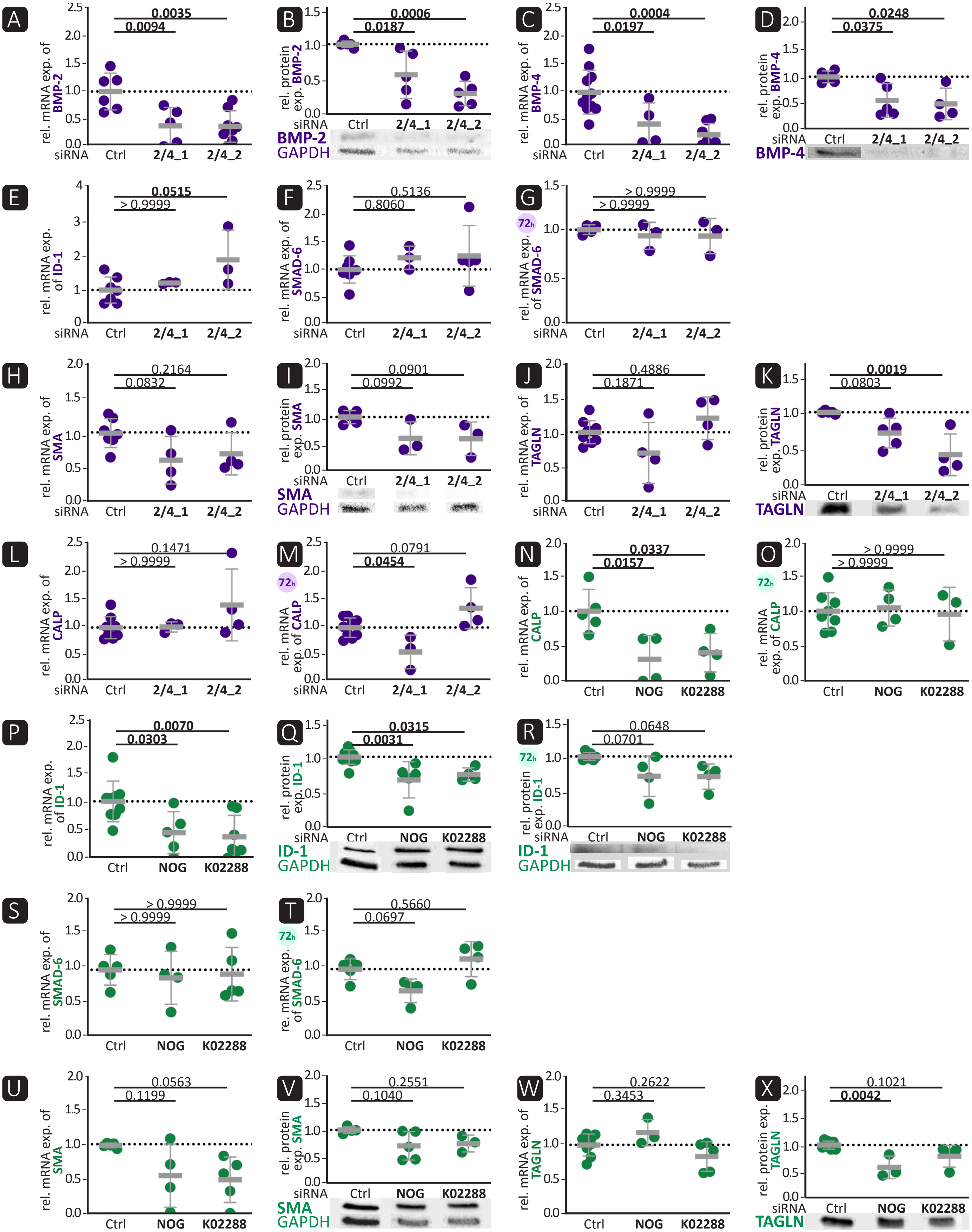
A
0.0035
0.0094
rel. mRNA exp. of BMP-2
siRNA Ctrl 2/4_1 2/4_2
B
0.0006
0.0187
rel. protein exp. BMP-2
siRNA Ctrl 2/4_1 2/4_2
BMP-2
GAPDH
C
0.0004
0.0197
rel. mRNA exp. of BMP-4
siRNA Ctrl 2/4_1 2/4_2
D
0.0248
0.0375
rel. protein exp. BMP-4
siRNA Ctrl 2/4_1 2/4_2
BMP-4
E
0.0515
> 0.9999
rel. mRNA exp. of ID-1
siRNA Ctrl 2/4_1 2/4_2
F
0.5136
0.8060
rel. mRNA exp. of SMAD-6
siRNA Ctrl 2/4_1 2/4_2
G
72h
> 0.9999
> 0.9999
rel. mRNA exp. of SMAD-6
siRNA Ctrl 2/4_1 2/4_2
H
0.2164
0.0832
rel. mRNA exp. of SMA
siRNA Ctrl 2/4_1 2/4_2
I
0.0901
0.0992
rel. protein exp. SMA
siRNA Ctrl 2/4_1 2/4_2
SMA
GAPDH
J
0.4886
0.1871
rel. mRNA exp. of TAGLN
siRNA Ctrl 2/4_1 2/4_2
K
0.0019
0.0803
rel. protein exp. TAGLN
siRNA Ctrl 2/4_1 2/4_2
TAGLN
L
0.1471
> 0.9999
rel. mRNA exp. of CALP
siRNA Ctrl 2/4_1 2/4_2
M
72h
0.0791
0.0454
rel. mRNA exp. of CALP
siRNA Ctrl 2/4_1 2/4_2
N
0.0337
0.0157
rel. mRNA exp. of CALP
Ctrl NOG K02288
O
72h
> 0.9999
> 0.9999
rel. mRNA exp. of CALP
Ctrl NOG K02288
P
0.0070
0.0303
rel. mRNA exp. of ID-1
Ctrl NOG K02288
Q
0.0315
0.0031
rel. protein exp. ID-1
siRNA Ctrl NOG K02288
ID-1
GAPDH
R
72h
0.0648
0.0701
rel. protein exp. ID-1
siRNA Ctrl NOG K02288
ID-1
GAPDH
S
> 0.9999
> 0.9999
rel. mRNA exp. of SMAD-6
Ctrl NOG K02288
T
72h
0.5660
0.0697
re. mRNA exp. of SMAD-6
Ctrl NOG K02288
U
0.0563
0.1199
rel. mRNA exp. of SMA
Ctrl NOG K02288
V
0.2551
0.1040
rel. protein exp. SMA
siRNA Ctrl NOG K02288
SMA
GAPDH
W
0.2622
0.3453
rel. mRNA exp. of TAGLN
Ctrl NOG K02288
X
0.1021
0.0042
rel. protein exp. TAGLN
siRNA Ctrl NOG K02288
TAGLN

Figure S9: Supplementary data to figure 6 and 7. VSMC were either transfected with a combination of BMP-2 and BMP-4 targeting siRNA (2/4_1, 2/4_2) compared to scrambled siRNA (Ctrl) as control or incubated with BMP inhibitors noggin (NOG; 100ng/mL) or K02288 (1 µM) in 0.4% FBS/EBM (Ctrl). Data are presented as mean±SD and comparisons were calculated by an ordinary one-way ANOVA-test with Bonferroni correction for multiple testing; p-values as indicated. Rel.=relative; exp.=expression. (**A+C+E+F+G+H+J+L+M+N+N+O+P+S+T+U+W**) qRT-PCR of (A) BMP-2 (n≥5), (C) BMP-4 (n≥4), (E+P) ID-1 (n≥3), (F+G+S+T) SMAD-6 (n≥3), (H+U) SMA (n≥4), (J+W) TAGLN (n≥3), (L+M+N+O) CALP (n≥3). (**B+D+I+K+Q+R+V+X**) Western blot analysis of (B) BMP-2 (n≥5), (D) BMP-4 (n≥4, representative blots of GAPDH shown in B), (I+V) SMA (n≥3), (K+X) TAGLN (n≥3; representative blots of GAPDH shown in I, V), (Q+R) ID-1 (n≥4) with GAPDH as loading control.

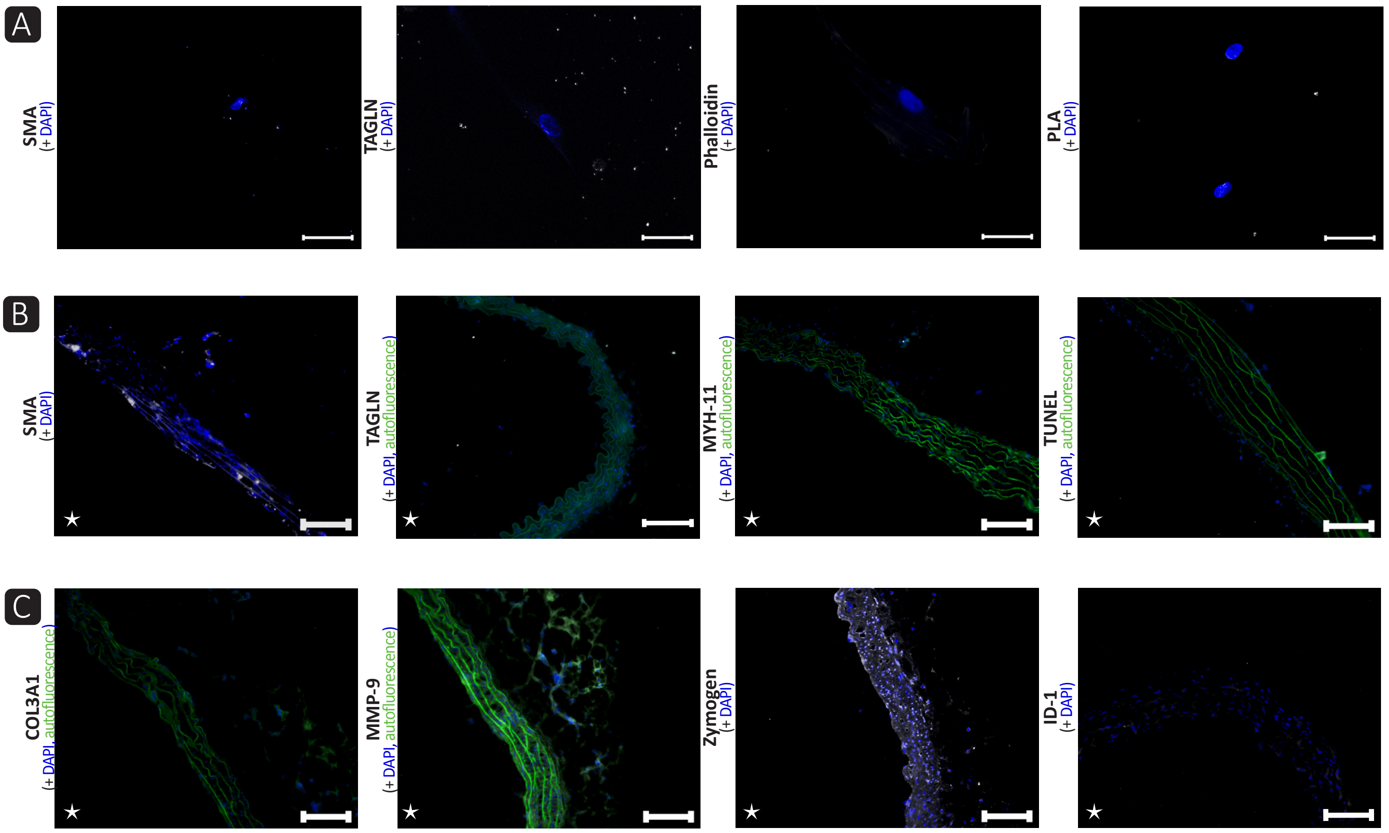
A
SMA (+ DAPI)
TAGLN (+ DAPI)
Phalloidin (+ DAPI)
PLA (+ DAPI)
B
SMA (+ DAPI)
TAGLN (+ DAPI, autofluorescence)
MYH-11 (+ DAPI, autofluorescence)
TUNEL (+ DAPI, autofluorescence)
C
COL3A1 (+ DAPI, autofluorescence)
MMP-9 (+ DAPI, autofluorescence)
Zymogen (+ DAPI)
ID-1 (+ DAPI)

Figure S10: Negative controls of immunocytological and immunohistological stainings. To investigate the background staining, a non-specific antibody at the same concentration was used instead of the target-specific antibody. Otherwise, the same protocol was used for the stainings and for image acquisition. The basal control condition was stained in each case. Nuclei were stained with DAPI (blue). Autofluorescence of elastic fibers in green when indicated in the figure. Vascular lumen is marked with an asterisk.(**A**) Representative photomicrographs of negative controls for immunocytological staining against alpha smooth muscle actin (SMA), transgelin (TAGLN), phalloidin and negative controls of *in-situ* proximity ligation assay (PLA) for the detection of BMPR1a phosphorylation. Scale bars, 50 μm. (**B**) Representative photomicrographs of negative controls of immunohistological staining of thoracic aortic tissue against SMA, TAGLN and myosin heavy chain 11 (MYH-11) and TUNEL assay. Scale bar 100 μm. (**C**) Representative photomicrographs of negative controls of immunohistological staining of thoracic aortic tissue against collagen3A1 (COL3A1), matrix metalloproteinase 9 (MMP-9), background activity of Zymogen assay and ID-1. Scale bar 100 μm.

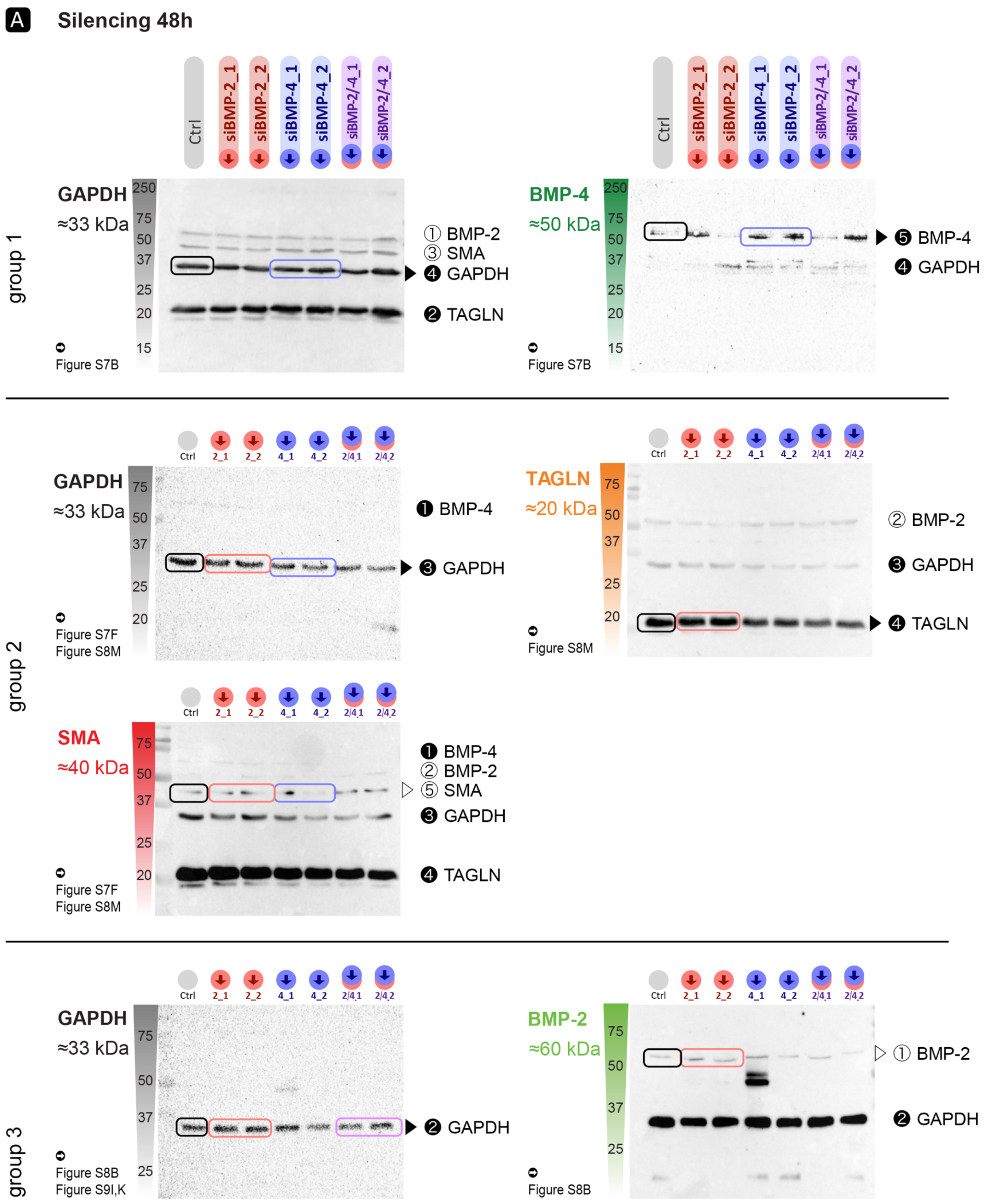
A
Silencing 48h
group 1
Ctrl
siBMP-2_1
siBMP-2_2
siBMP-4_1
siBMP-4_2
siBMP-2/-4_1
siBMP-2/-4_2
GAPDH
≈33 kDa
250
75
50
37
25
20
15
Figure S7B
① BMP-2
③ SMA
❹ GAPDH
❷ TAGLN
BMP-4
≈50 kDa
Figure S7B
❺ BMP-4
❹ GAPDH
group 2
Ctrl
2_1
2_2
4_1
4_2
2/4 1
2/4 2
GAPDH
≈33 kDa
Figure S7F
Figure S8M
❶ BMP-4
❸ GAPDH
TAGLN
≈20 kDa
Figure S8M
② BMP-2
❸ GAPDH
❹ TAGLN
SMA
≈40 kDa
Figure S7F
Figure S8M
❶ BMP-4
② BMP-2
⑤ SMA
❸ GAPDH
❹ TAGLN
group 3
GAPDH
≈33 kDa
Figure S8B
Figure S9I,K
❷ GAPDH
BMP-2
≈60 kDa
Figure S8B
① BMP-2
❷ GAPDH

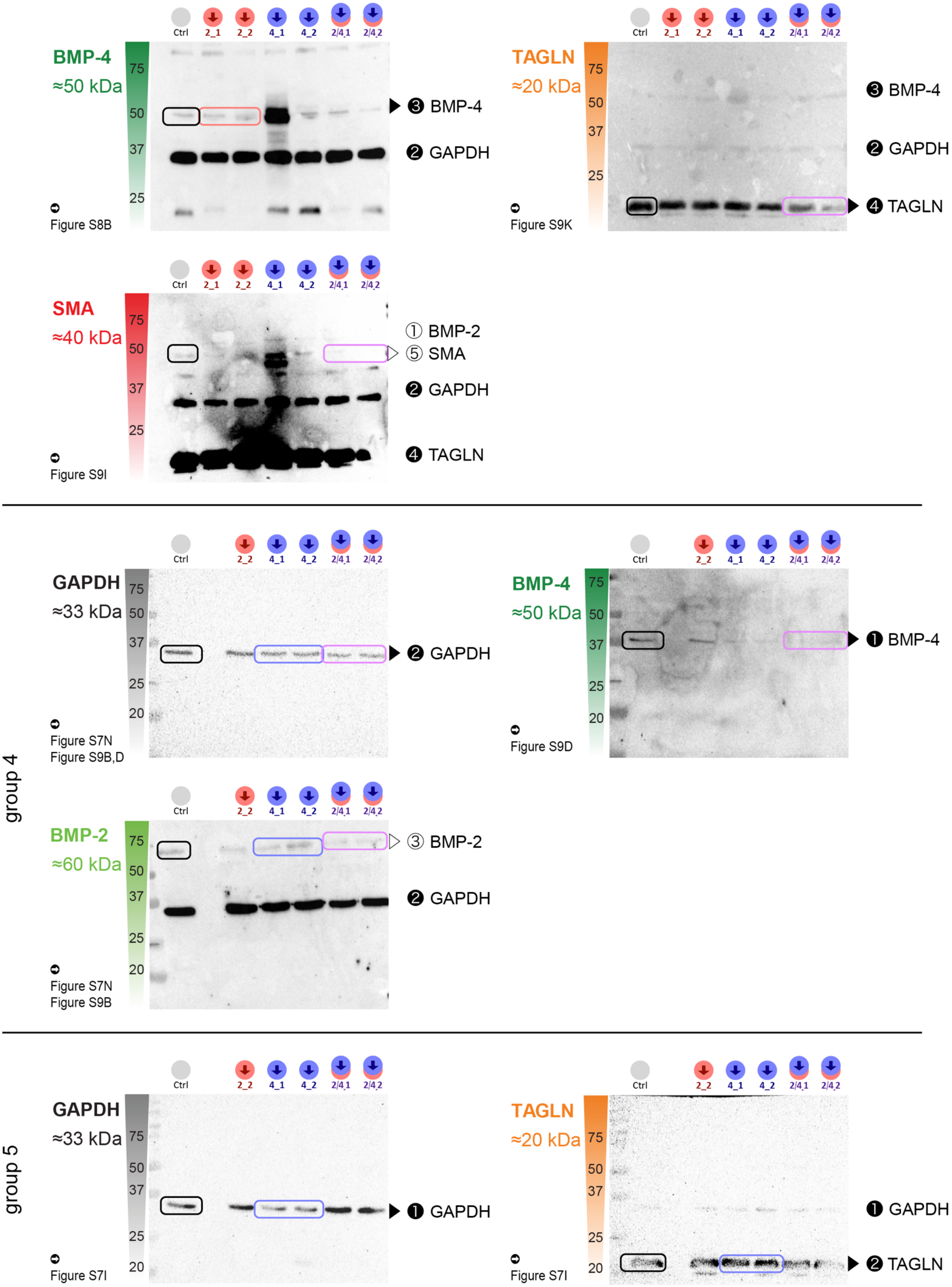

Ctrl
2_1
2_2
4_1
4_2
2/4.1
2/4.2
BMP-4
≈50 kDa
75
50
37
25
❸ BMP-4
❷ GAPDH
Figure S8B
TAGLN
≈20 kDa
❸ BMP-4
❷ GAPDH
❹ TAGLN
Figure S9K
SMA
≈40 kDa
① BMP-2
⑤ SMA
❷ GAPDH
❹ TAGLN
Figure S9I
group 4
GAPDH
≈33 kDa
20
❷ GAPDH
Figure S7N
Figure S9B,D
BMP-4
≈50 kDa
❶ BMP-4
Figure S9D
BMP-2
≈60 kDa
③ BMP-2
❷ GAPDH
Figure S7N
Figure S9B
group 5
GAPDH
≈33 kDa
❶ GAPDH
Figure S7I
TAGLN
≈20 kDa
❶ GAPDH
❷ TAGLN
Figure S7I

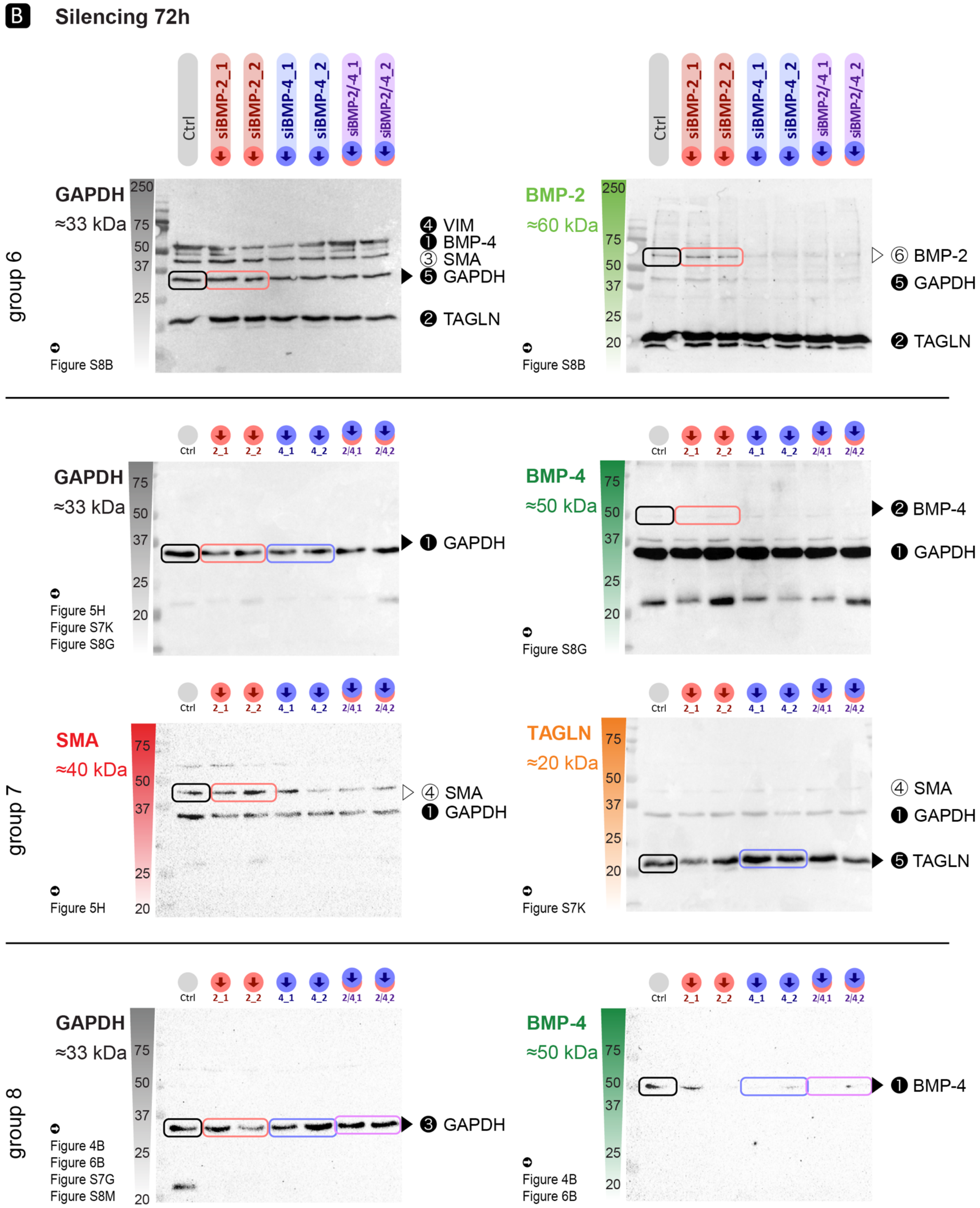
B
Silencing 72h
group 6
Ctrl
siBMP-2_1
siBMP-2_2
siBMP-4_1
siBMP-4_2
siBMP-2/4_1
siBMP-2/4_2
GAPDH
≈33 kDa
250
75
50
37
25
❹ VIM
❶ BMP-4
③ SMA
❺ GAPDH
❷ TAGLN
Figure S8B
BMP-2
≈60 kDa
250
75
50
37
25
20
⑥ BMP-2
❺ GAPDH
❷ TAGLN
Figure S8B
Ctrl
2_1
2_2
4_1
4_2
2/4 1
2/4 2
GAPDH
≈33 kDa
75
50
37
25
20
❶ GAPDH
Figure 5H
Figure S7K
Figure S8G
BMP-4
≈50 kDa
75
50
37
25
20
❷ BMP-4
❶ GAPDH
Figure S8G
group 7
SMA
≈40 kDa
75
50
37
25
20
④ SMA
❶ GAPDH
Figure 5H
TAGLN
≈20 kDa
75
50
37
25
20
④ SMA
❶ GAPDH
❺ TAGLN
Figure S7K
group 8
GAPDH
≈33 kDa
75
50
37
25
20
❸ GAPDH
Figure 4B
Figure 6B
Figure S7G
Figure S8M
BMP-4
≈50 kDa
75
50
37
25
20
❶ BMP-4
Figure 4B
Figure 6B

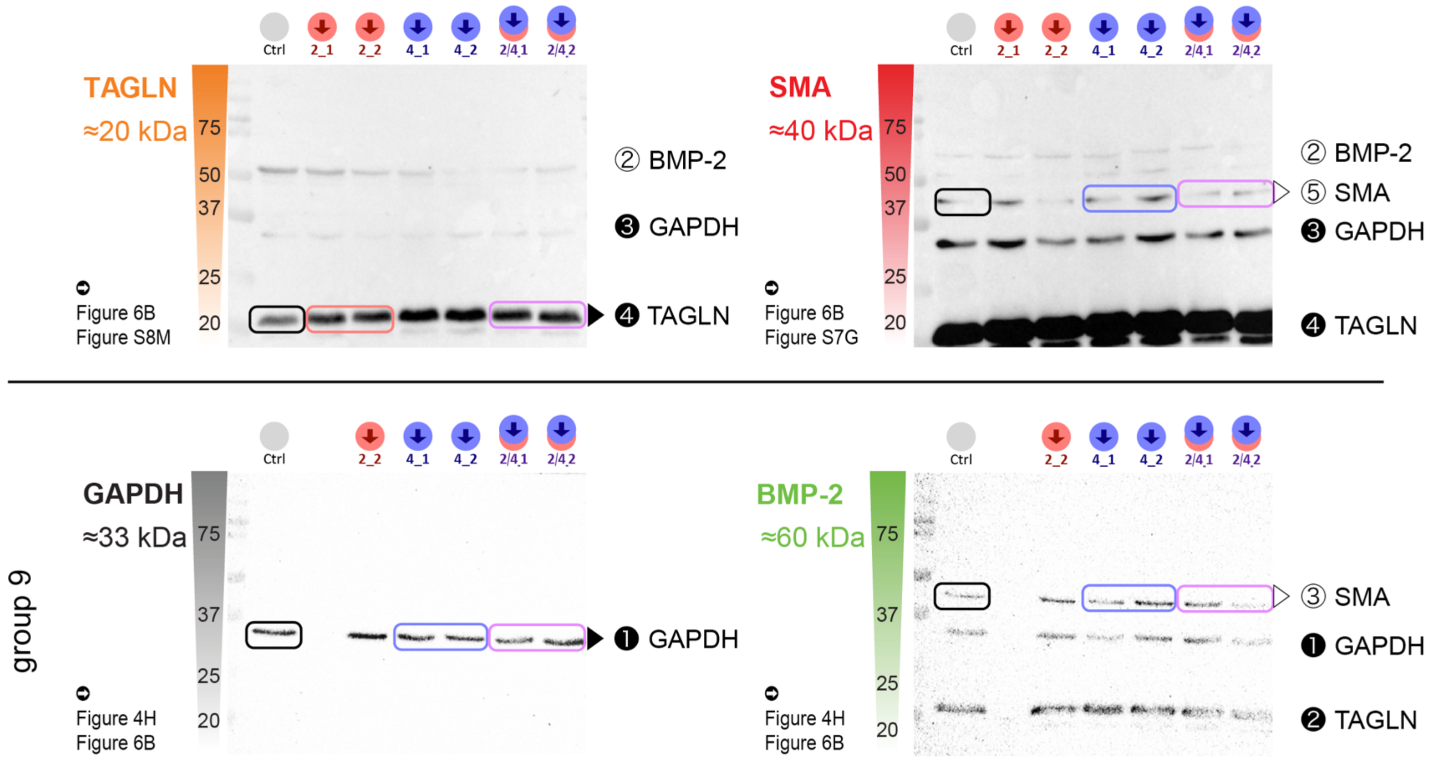

C Stimulation / Inhibition 48h

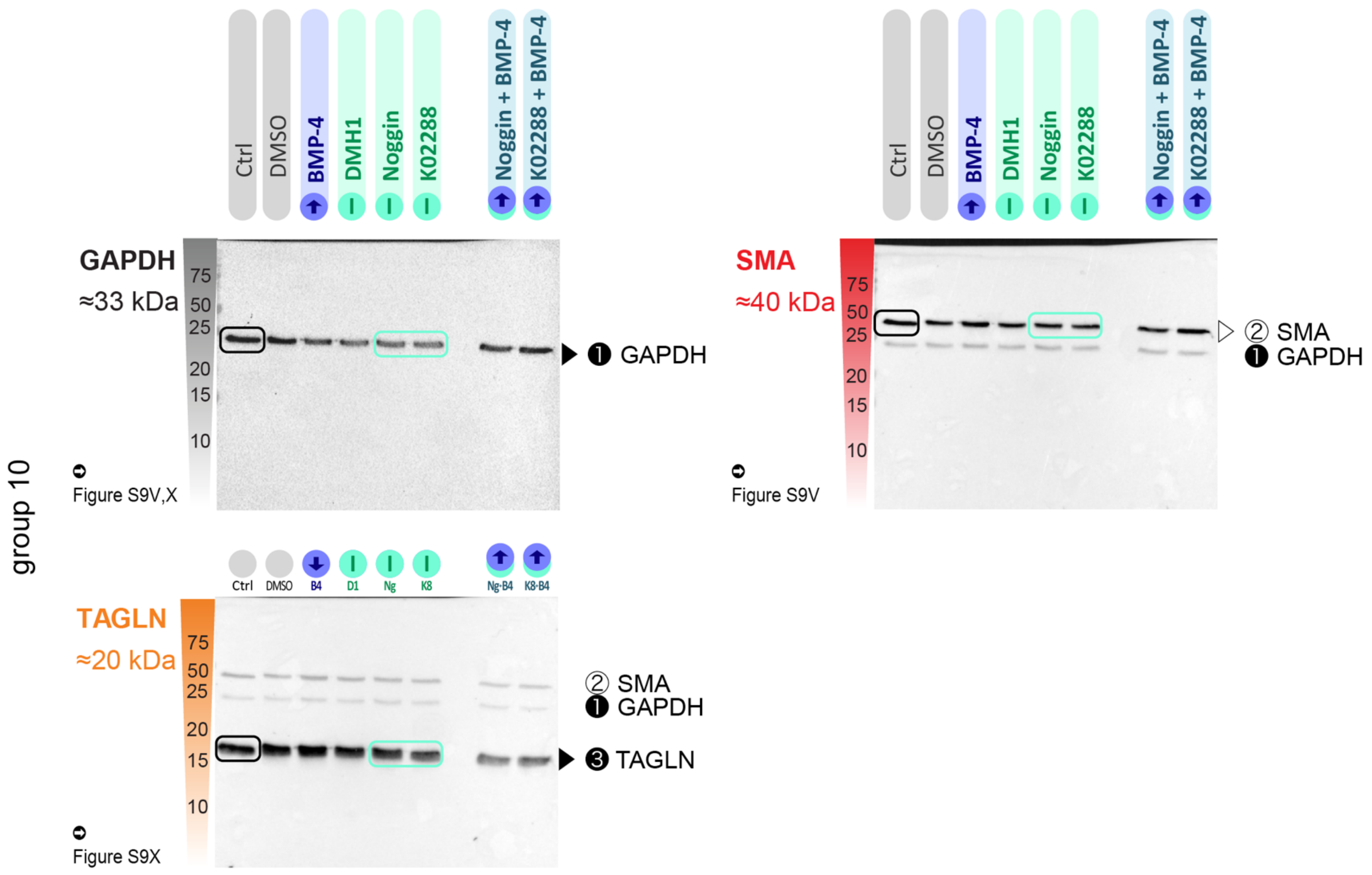

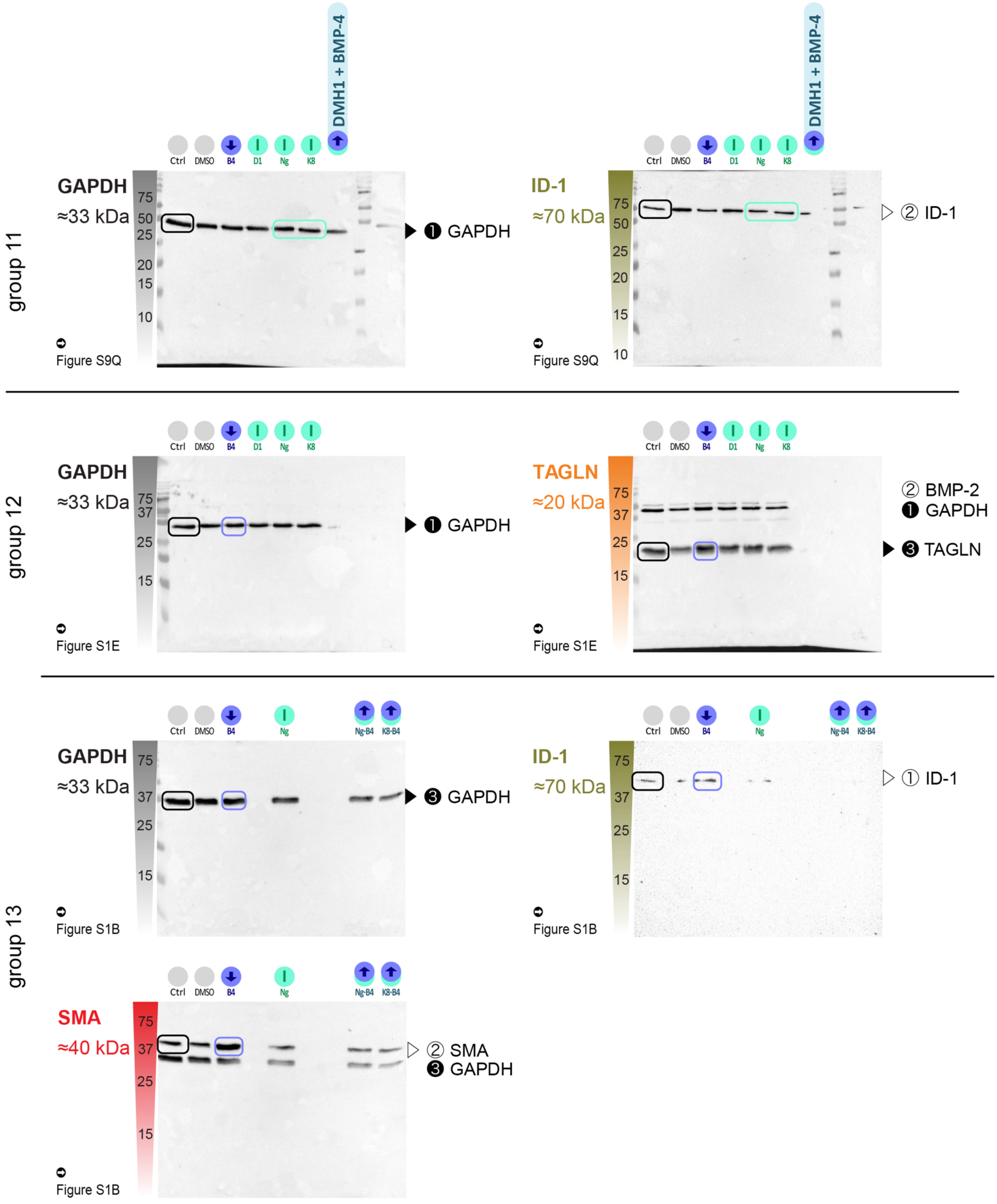
group 11
DMH1 + BMP-4
Ctrl DMSO B4 D1 Ng K8
GAPDH
≈33 kDa
75 50 25 20 15 10
❶ GAPDH
Figure S9Q
ID-1
≈70 kDa
75 50 25 20 15 10
② ID-1
Figure S9Q
group 12
Ctrl DMSO B4 D1 Ng K8
GAPDH
≈33 kDa
75 37 25 15
❶ GAPDH
Figure S1E
TAGLN
≈20 kDa
75 37 25 15
② BMP-2
❶ GAPDH
❸ TAGLN
Figure S1E
group 13
Ctrl DMSO B4 Ng Ng-B4 K8-B4
GAPDH
≈33 kDa
75 37 25 15
❸ GAPDH
Figure S1B
ID-1
≈70 kDa
75 37 25 15
① ID-1
Figure S1B
SMA
≈40 kDa
75 37 25 15
② SMA
❸ GAPDH
Figure S1B

D Stimulation / Inhibition 72h

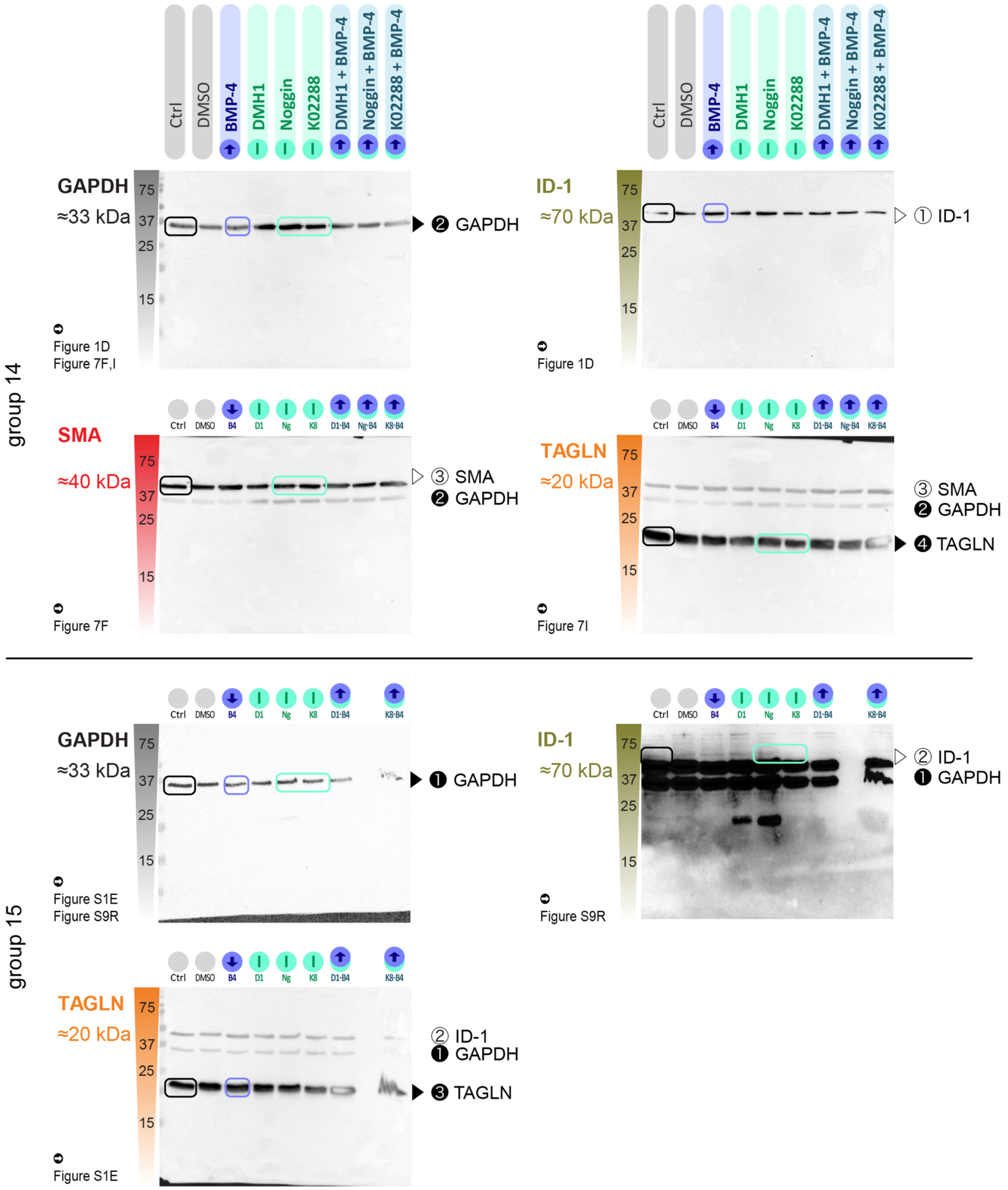

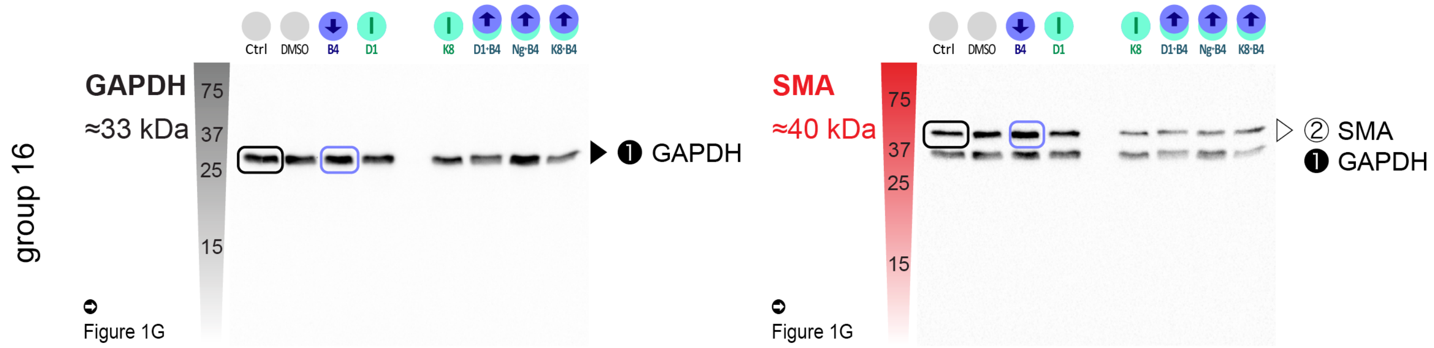

E **Murine proteins**

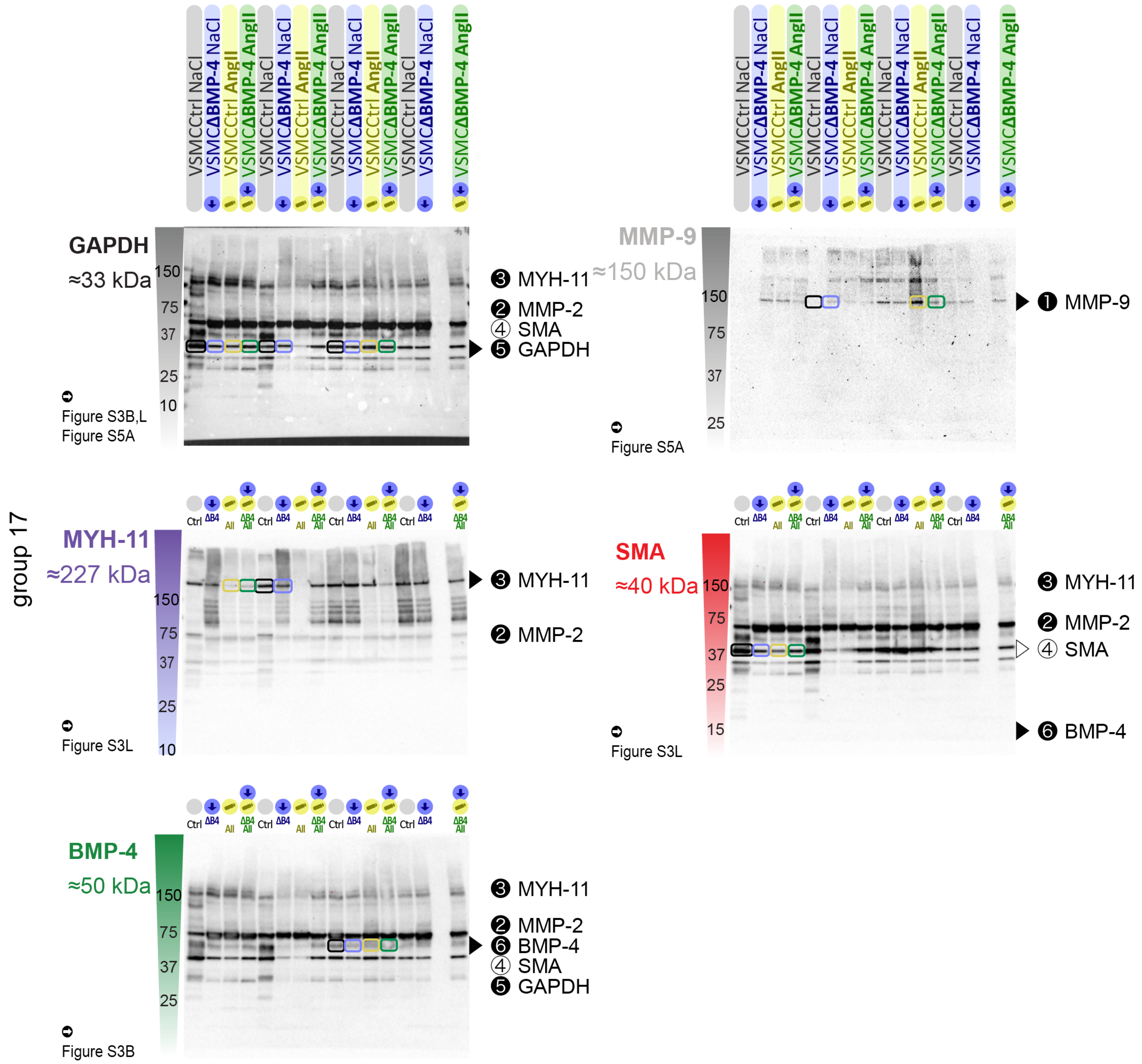

Figure S11 Full unedited western blots. The same loading scheme was used for all gels of a subpanel (siBMP-2_1 = 2_1, siBMP-2_2 = 2_2, siBMP-4_1 = 4_1, siBMP-4_2 = 4_2, BMP-4 = B4, DMH1 = D1, Noggin = Ng, K02288 = K8, VSCMCtrl NaCl = Ctrl VSMCΔBMP-4 NaCl = ΔB4, VSMCCtrl AngII = AII, VSMCΔBMP-4 AngII = B4+AII). The antibodies that led to the detection of the corresponding bands and their order are indicated on the right of each image. Numbers with black background are rabbit antibodies, numbers with white background are mouse antibodies. The membrane used for analysis after incubation with the antibody of interest is indicated by an arrow in front of the number and the relevant sections are marked. In addition, antibodies against vimentin (VIM) and MMP-2 are visible on some of the membranes, but these were not analysed any further. An approximate scale is always given to the left of the membrane for reference. The bands have been identified based on their size, the order of the antibodies used and the type of secondary antibody used.

MAJOR RESOURCES TABLE

Animals (*in-vivo* studies)

Species	Vendor or Source	Background Strain	Sex	Persistent ID/URL
Mus musculus	Charles River	C57/BL6N	male	www.criver.com/products-services/find-model/c57bl6-mouse?region=23

Genetically Modified Animals

	Species	Vendor or Source	Background Strain	Persistent ID / URL
B6.129BS-Bmp4^{tm4Blh}	Mus musculus	Bridgid Hogan's lab	C57/BL6N	www.cellbio.duke.edu/primary-faculty/brigid-lm-hogan
B6.Tg(MYH11-icre/ERT2)^{1Soff}	Mus musculus	The Jackson Laboratory	C57/BL6N	www.jax.org/strain/019079

Antibodies Immunhistology

Target antigen	Vendor or Source	Catalog #	Working concentration	Persistent ID / URL
Anti-Actin α-Smooth muscle monoclonal mouse FITC conjugated	Sigma-Aldrich, Schnelldorf, Germany	F3777	1:300	www.sigmaaldrich.com/AT/de/product/sigma/f3777
Anti-SMHC monoclonal rabbit	Abcam®, Cambridge, UK	ab124679	1:200	www.abcam.com/smooth-muscle-myosin-heavy-chain-11-antibody-epr5335-ab124679.htmL
Anti-Transgelin polyclonal rabbit	Abcam®, Cambridge, UK	ab14106	1:200	www.abcam.com/tagln-transgelin-antibody-ab14106.htmL
Anti-Collagen III polyclonal rabbit	Abcam®, Cambridge, UK	ab7778	1:200	www.abcam.com/collagen-iii-antibody-ab7778.htmL
Anti-MMP-9 polyclonal rabbit	Abcam®, Cambridge, UK	ab38898	1:200	www.abcam.com/mmp9-antibody-ab38898.htmL
Anti-ID-1 polyclonal rabbit	Abcam®, Cambridge, UK	ab203202	1:100	www.abcam.com/id1-antibody-ab203202.htmL
Negative control mouse IgG2A-FITC	Sigma-Aldrich, Schnelldorf, Germany	MABC004F	-	https://www.sigmaaldrich.com/AT/de/product/mm/mabc004f

Negative control rabbit IgG	Dako Cytomation, Hamburg, Germany	X0936	-	-
Alexa Fluor 555 donkey anti-rabbit IgG (H+L)	Thermo Fisher Scientific, Schwerte, Germany	A-31572	1:500	www.thermofisher.com/antibody/product/Donkey-anti-Rabbit-IgG-H-L-Highly-Cross-Adsorbed-Secondary-Antibody-Polyclonal/A-31572

Antibodies Immuncytology

Target antigen	Vendor or Source	Catalog #	Working concentration	Persistent ID / URL
Anti-Actin α-Smooth muscle monoclonal mouse FITC conjugated	Sigma-Aldrich, Schnelldorf, Germany	F3777	1:300	www.sigmaaldrich.com/AT/de/product/sigma/f3777
Anti-Transgelin polyclonal rabbit	Abcam®, Cambridge, UK	ab14106	1:100	www.abcam.com/tagln-transgelin-antibody-ab14106.htmL
Negative control mouse IgG2A-FITC	Sigma-Aldrich, Schnelldorf, Germany	MABC004F	-	www.sigmaaldrich.com/AT/de/product/mm/mabc004f
Negative control rabbit IgG	Dako Cytomation, Hamburg, Germany	X0936	-	-
Alexa Fluor 555 donkey anti-rabbit IgG (H+L)	Thermo Fisher Scientific, Schwerte, Germany	A-31572	1:400	www.thermofisher.com/antibody/product/Donkey-anti-Rabbit-IgG-H-L-Highly-Cross-Adsorbed-Secondary-Antibody-Polyclonal/A-31572

Antibodies western blot

Target antigen	Vendor or Source	Catalog #	Working concentration	Persistent ID / URL
Anti-Actin α-Smooth muscle monoclonal mouse	Sigma-Aldrich, Schnelldorf, Germany	A 2547	1:5000	www.sigmaaldrich.com/AT/de/product/sigma/f3777
Anti-SMHC monoclonal rabbit	Abcam®, Cambridge, UK	ab124679	1:10000	www.abcam.com/smooth-muscle-myosin-heavy-chain-11-antibody-epr5335-ab124679.htmL
Anti-Transgelin polyclonal rabbit	Abcam®, Cambridge, UK	ab14106	1:2000	www.abcam.com/tagln-transgelin-antibody-ab14106.htmL
Anti-MMP-9 polyclonal rabbit	Abcam®, Cambridge, UK	ab38898	1:1000	www.abcam.com/mmp9-antibody-ab38898.htmL
Anti-ID-1 polyclonal rabbit	Abcam®, Cambridge, UK	ab203202	1:1000	www.abcam.com/id1-antibody-ab203202.htmL

Anti-BMP-2 monoclonal mouse	R&D Systems, Wiesbaden, Germany	MAB3551	1:500	www.rndsystems.com/products/human-bmp-2-antibody-100221_mab3551
Anti-BMP-4 polyclonal rabbit	ThermoFisher Scientific, Schwerte, Germany	PA5-19683	1:1000	www.thermofisher.com/antibody/product/BMP-4-Antibody-Polyclonal/PA5-19683
Anti-GAPDH polyclonal rabbit	EnoGene Biotech, New York, USA	E1C604	1:5000	www.enogene.com/product/E1C604-1
Anti-mouse IgG/HRP conjugated	R&D Systems, Wiesbaden, Germany	HAF007	1:5000	www.rndsystems.com/products/mouse-igg-hrp-conjugated-antibody_haf007
Anti-rabbit IgG/HRP conjugated	ThermoFisher Scientific, Schwerte, Germany	31460	1:10000	www.thermofisher.com/antibody/product/Goat-anti-Rabbit-IgG-H-L-Secondary-Antibody-Polyclonal/31460

Recombinant proteins

Target antigen	Vendor or Source	Catalog #	Working concentration	Lot # (preferred but not required)	Persistent ID / URL
Recombinant human BMP-4	R&D Systems, Wiesbaden, Germany	314-BP-010	50 ng/mL	BEM9514081	www.rndsystems.com/products/recombinant-human-bmp-4-protein_314-bp
Recombinant human Noggin	R&D Systems, Wiesbaden, Germany	6057-NG-025	100 ng/mL	TNT2820061	https://www.rndsystems.com/products/recombinant-human-noggin-protein_6057-ng
K02288	TOCRIS, Bristol, UK	4986	1µM	1	https://www.tocris.com/products/k-02288_4986

siRNA

siRNA	Clone Name	Sequence	Source / Repository
2_1	BMP2HSS101044	(RNA)-GGA AAC GCC UUA AGU CCA GCU GUA A<br>(RNA)-UUA CAG CUG GAC UUA AGG CGU UUC C	Thermo Fisher Scientific, Schwerte, Germany
2_2	BMP2HSS101045	(RNA)-GGU CAA CUC UGU UAA CUC UAA GAU U<br>(RNA)-AAU CUU AGA GUU AAC AGA GUU GAC C	
4_1	BMP4HSS101052	(RNA)-GCU UCC ACC GUA UAA ACA UUU AUG A<br>(RNA)-UCA UAA AUG UUU AUA CGG UGG AAG C	
4_2	BMP4HSS101050	(RNA)-GCQ UGU CAG GAU UAG CCG AUC GUU A<br>(RNA)-UAA CGA UCG GCU AAU CCU GAC AUG C	

Primer for qRT-PCR

Clone Name	Sequence	Source / Repository	Persistent ID / URL
ID-1 (human)	Fwd: 5′-CTACGACATGAACGGCTGCTACTC-3′<br>Rev: 5′- GTAGAGCAGGACGTTCACCT-3′		eurofinsgenomics.com

ID-1 (murine)	Fwd: 5′- CCTAGCTGTTCGCTGAAGGC-3′<br>Rev: 5′-CTTGCTCACTTTGCGGTTCT-3′	Eurofins MWG Operon, Ebersberg, Germany	
SMA (human)	Fwd: 5′-CCCAGACATCAGGGAGTAATG-3′<br>Rev: 5′-TCTATCGGATACTTCAGCGTC-3′		
SMA (murine)	Fwd: 5′- CCCAGACATCAGGGAGTAATGG-3′<br>Rev: 5′- TCTATCGGATACTTCAGCGTCA-3′		
TAGLN (human)	Fwd: 5′- ACCAAAAACGATGGAAACTACCG-3′<br>Rev: 5′- GTGAAGTCCCTCTTATGFTCCT-3′		
TAGLN (murine)	Fwd: 5′-AACAGCCTGTACCCTGATGG-3′<br>Rev: 5′-CGGTAGTGCCCATCATTCTT-3′		
MYH-11	Fwd: 5′- AAGCTGCGGCTAGAGGTCA-3′<br>Rev: 5′- CCCTCCCTTTGATGGCTGAG-3′		
CALP	Fwd: 5′-AGCTAAGAGAAGGGCGGAAC-3′<br>Rev: 5′-CATCTGCAGGCTGACATTGA-3′		
Col3A1	Fwd: 5′-GCCAAATATGTGTCTGTGACTCA-3′<br>Rev: 5′-GGGCGAGTAGGAGCAGTTG-3′		
MMP-9	Fwd: 5′-CCTGGAGACCTGAGAACCAATC-3′<br>Rev: 5′-CCACCCGAGTGTAACCATAGC-3′		
BMP-2	Fwd: 5′-AACACTGTGCGCAGCTTCC-3′<br>Rev: 5′-CTCCGGGTTGTTTTCCCAC-3′		
BMP-4	Fwd: 5′-CACGAAGAACATCTGGAGAAC-3′<br>Rev: 5′-CCCTTGAGGTAACGATCAGCT-3′		
SMAD-6	Fwd: 5′-CCCCCGGCTACTCCATCAAGGTGT-3′<br>Rev: 5′-GTCCGTGGGGGCTGTGTCTCTGG-3′		
hRPII	Fwd: 5′-GCACCACGTCCAATGACAT-3′<br>Rev: 5′-GTGCGGCTGCTTCCATAA-3′		
M36B4	Fwd: 5′-AAGCGCGTCCTGGCATTGTCT-3′<br>Rev: 5′-CCGCAGGGGCAGCAGTGGT-3′		

Cultured Cells

**Name**	**Vendor or Source**	**Sex (F, M, or unknown)**	**Persistent ID / URL**
Human Aortic Smooth Muscle Cells	PELO biotech	Unknown	Catalog No: PB-CH-280-2011

Software

**Software**	**Source / Repository**
GraphPad Prism 9	GraphPad Software, La Jolla, US-CA
Microsoft Word Version 16	Microsoft Corporation, Redmond, USA
Bio-Rad CFX Manager Version 3.1	Bio-Rad GmbH, Munich, Germany
Image Lab Version 4	Bio-Rad GmbH, Munich, Germany
ZEN 3.1 blue edition	Carl Zeiss GmbH, Jena, Germany
Pixelmator Pro Version 2	Pixelmator Team, Vilnius, Lithuania
Affinity Designer 2	Serif Nottingham, UK

Affinity Publisher 2	Serif Nottingham, UK
ImageJ2 Version 2.3.0/1.53q	Open Source
Matlab script Version 9.6.0.1214997	MatWorks Inc, Natick, USA

ARRIVE GUIDELINES

Study Design

Groups	Sex	Age	Number (prior to experiment)	Number (after termination)	Littermates (Yes/No)	Other description
MYH11Cre-BMP4flox TAM- NaCl	male	18-21 weeks	18	17	Yes	Pump implantation, Histology qRT-PCR, western Blot
MYH11Cre-BMP4flox TAM+ NaCl	male	18-21 weeks	19	19	Yes	Pump implantation, Histology qRT-PCR, western Blot
MYH11Cre-BMP4flox TAM- AngII	male	18-21 weeks	19	17	Yes	Pump implantation, Histology qRT-PCR, western Blot
MYH11Cre-BMP4flox TAM+AngII	male	18-21 weeks	20	20	Yes	Pump implantation, Histology qRT-PCR
MYH11Cre-BMP4flox TAM-	male	7-9 weeks	15	15	Yes	Nanoindentation, Histology, qRT-PCR,
MYH11Cre-BMP4flox TAM+	male	7-9 weeks	16	16	Yes	Nanoindentation, Histology, qRT-PCR
MYH11Cre TAM-	male	6-9 weeks	8	8	Yes	Histology
MYH11Cre TAM+	male	6-9 weeks	9	9	Yes	Histology

Sample Size:
Given the investigator's assumed standardised effect size of 1.06, a desired power of 80%, and a two-sided significance level of 5%, a sample size of at least 7-15 animals per method and group is required to detect a statistically significant difference using the unpaired t-test, depending on the method used. A statistical assessment was also performed.

Inclusion Criteria
Only healthy male animals with inconspicuous behaviour at the age of 6 or 24 weeks were included in the experiment.

Exclusion Criteria
In the presence of conditions that may affect the outcome of the test or in the presence of severe functional impairment or symptoms of disease, the test should be terminated. For this purpose, the animals were observed daily for the first 48 h postoperatively. Withdrawal criteria include a 20% decrease in body weight from baseline, convulsions, paralysis (trunk muscles, extremities), respiratory noises, tremors and ataxia. If there is any doubt about an animal's distress, the animal welfare officer will be consulted immediately. Outside normal working hours, the experiment will be stopped in case of doubt. The animals are killed by cervical dislocation.

Randomization
The allocation of animals to each experimental group was randomised on the basis of the randomly assigned animal numbers.

Blinding
Data were analysed without knowledge of genotype and treatment and assigned to groups only after analysis using the corresponding animal number.
